# Carrier States in Ferromagnetic Semiconductors and Diluted Magnetic Semiconductors—Coherent Potential Approach—

**DOI:** 10.3390/ma3063740

**Published:** 2010-06-21

**Authors:** Masao Takahashi

**Affiliations:** Kanagawa Institute of Technology, 1030 Shimo-Ogino, Atsugi, 243-0292, Japan; E-Mail: taka@gen.kanagawa-it.ac.jp; Fax: +81-46-291-3107

**Keywords:** magnetic semiconductor, coherent potential approximation (CPA), exchange interaction, carrier-induced ferromagnetism

## Abstract

The theoretical study of magnetic semiconductors using the dynamical coherent potential approximation (dynamical CPA) is briefly reviewed. First, we give the results for ferromagnetic semiconductors (FMSs) such as EuO and EuS by applying the dynamical CPA to the *s*-*f* model. Next, applying the dynamical CPA to a simple model for A${}_{1-x}$Mn${}_{x}$B-type diluted magnetic semiconductors (DMSs), we show the results for three typical cases to clarify the nature and properties of the carrier states in DMSs. On the basis of this model, we discuss the difference in the optical band edges between II-V DMSs and III-V-based DMSs, and show that two types of ferromagnetism can occur in DMSs when carriers are introduced. The carrier-induced ferromagnetism of Ga${}_{1-x}$Mn${}_{x}$As is ascribed to a double-exchange (DE)-like mechanism realized in the magnetic impurity band/or in the band tail.

## 1. Introduction

For more than three decades, magnetic semiconductors have attracted much attention because of their combination of magnetic and semiconducting properties.

In the 1960s and 1970s, many papers were published on ferromagnetic semiconductors (FMSs) such as EuO and EuS [1,2]. Pure EuO and EuS are considered as typical Heisenberg ferromagnets. Their magnetic moments originate from the half-filled and highly localized 4f shell of the Eu ion at regular sites. At the high-temperature limit (T=∞), the orientation of each localized spin (*f* spin) is completely random. At paramagnetic temperatures the total magnetization is zero, although short-range order is formed as the temperature decreases to the Curie temperature $\left(T_{c}\right)$. Below the Curie temperature, spontaneous magnetic ordering develops, and at T=0 the orientations of *f* spins are perfectly arranged in one direction. Eu chalcogenides are also insulators with a NaCl-type structure. When a single electron is introduced into the crystal, the electron enters an otherwise empty (5d,6s) conduction band. The electron (hereafter referred to as the *s* electron) moves in the crystal while interacting with *f* spins through the *s*-*f* exchange interaction. Thus, the conduction- (*s*-) electron state in FMSs is strongly affected by the magnetic order of *f* spins. This causes numerous anomalous phenomena in FMSs, such as the redshift of the optical absorption edge with decreasing temperature, the magnetic polaron effect, spin-disorder scattering around $T_{c}$ [3], and the metal-insulator transition in Eu-rich EuO [4]. Its extreme properties, such as high electron-spin polarization, colossal magnetic resistance (CMR), and the enhancement of $T_{c}$ due to Gd doping, make electron-doped EuO interesting for spintronics applications. Recently, these features have therefore stimulated systematic experimental studies with modern techniques and improved sample quality [5,6,7,8,9,10,11,12,13] as well as theoretical studies [14,15,16,17,18]. The conduction-electron states in FMSs have been discussed on the basis of the *s*-*f* (exchange) model [19,20]; the *s*-*f* model is sometimes referred to as the Kondo lattice model [21,22].

Diluted magnetic semiconductors (DMSs) are semiconducting alloys whose lattice is partly made up of substitutional magnetic atoms. The most extensively studied DMSs since the 1980s are $A_{1-x}^{\mathrm{II}}$Mn${}_{x}B^{\mathrm{VI}}$-type (II-VI-based) DMSs, in which a fraction of the group II sublattice is replaced at random by magnetic Mn atoms. Mn impurities substituting for 2+ cations act as stable 2+ ions; therefore, there are few carriers, which makes these DMSs insulators. It is widely accepted that in II-VI-based DMSs the carriers (*s* electrons and *p* holes) move over many sites while interacting with the localized (*d*) spins at Mn sites through the *sp-d* exchange interaction [23,24,25]. The exchange interaction strongly enhances the effect of the magnetic field on band splitting, leading to spectacular magnetooptical effects (e.g., giant Faraday rotation or Zeeman splitting). Since 1996, attention has also been focused on the III-V-based DMSs of Ga${}_{1-x}$Mn${}_{x}$As and In${}_{1-x}$Mn${}_{x}$As owing to their high potential for new device applications. It is highly noteworthy that the doping of Mn into GaAs and InAs leads to ferromagnetism and interesting magneto-optical and magnetotransport phenomena. This ferromagnetism is generally referred to as “carrier-induced ferromagnetism” because hole carriers introduced by Mn incorporation mediate the ferromagnetic coupling between Mn ions [26,27]. The main difference between II-VI and III-V-based DMSs is that in the latter, Mn ions act not only as magnetic impurities but also as acceptors. The microscopic mechanism for carrier-induced ferromagnetism is still controversial. To elucidate the origin and mechanism of the carrier-induced ferromagnetism, however, it appears necessary to clarify the nature of the carrier states in DMSs.

The carrier states in FMSs and DMSs have not yet been explained theoretically in a sufficiently clear way. The exchange interaction between the carrier and magnetic moments (localized spin), however, seems to be a clue to solving most of these problems. Since the localized spins thermally fluctuate in FMSs, the theory should properly take into account the effect of the thermally fluctuating spins on carrier states through the exchange interaction. In DMSs, substitutional disorder exists in addition to the thermal fluctuation of localized spins at Mn sites.

The coherent potential approximation (CPA) is a superior mean-field theory which was originally devised for describing the electronic structure and/or the properties of binary substitutional alloys [28,29,30]. Rangette etal. first applied the CPA to the *s*-*f* model at high temperatures, assuming the orientations of the magnetic moments to be completely random [31]. In 1974 Kubo extended the CPA to thermally fluctuating spin systems; Kubo formulated the CPA in terms of effective locators [32]. In 1996 Takahashi etal. formulated the CPA in the *t*-matrix formula, which has been proved to be equivalent to the locator-formula CPA. Since then, the CPA has been applied to investigate FMSs [33,34,35,36,37] and/or DMSs [38,39,40,41,42,43]. This method is referred to as “dynamical CPA", because the dynamical spin-flip process is properly taken into account in the thermal averaging operation. In the classical spin limit, the numerical results obtained by dynamical CPA are in good agreement with those obtained by dynamical mean-field theory (DMFT) [44,45]. It has also been reported that the result for optical conductivity obtained by the dynamical CPA is in reasonable agreement with that obtained by Monte Carlo (MC) simulation [46]. Note that the dynamical CPA can be applied even for a finite magnitude of localized spin. Furthermore, the analytic formula in limiting cases, such as at high temperatures and at the diluted impurity limit, can be easily deduced.

In this article we briefly review the study of magnetic semiconductors using the dynamical CPA. We first discuss the conduction-electron states in FMSs on the basis of the *s*-*f* model. We formulate the dynamical CPA using multiple-scattering theory and discuss the numerical results. Next, we study the carrier states in II-VI and III-V-based DMSs using a simple model. The numerical results and discussion are first given for two typical cases with no nonmagnetic attractive potential: (i) the case of a strong exchange interaction and (ii) the case of a moderate exchange interaction. Since the screened Coulomb attractive potential acts between a hole and a Mn acceptor center in III-V-based DMSs, we also study (iii) the case of a moderate exchange interaction with a strong nonmagnetic attractive potential. Then, based on the Curie temperature $T_{c}$ numerically estimated in a simple way, we investigate the type and properties of the magnetism that may occur when carriers are introduced into DMSs. After that, we focus on (Ga,Mn)As, which has attracted much attention in recent years owing to its so-called carrier-induced ferromagnetism. Throughout the present review, we study the effect of the exchange interaction between the carrier and the localized spins on carrier states in magnetic semiconductors. In the Appendix, the locator formula of the dynamical CPA is briefly summarized.

## 2. Conduction-electron States in a Ferromagnetic Semiconductor (FMS)

### 2.1. Coherent Potential Approach to the s-f Model

The *s*-*f* (exchange) model is currently accepted as a basis for studying the conduction-electron states in an ordinary FMS such as EuO or EuS. In this model, there are magnetic moments at the regular sites and a well-defined conduction band. A single conduction electron (*s* electron) moves in the crystal while interacting with localized magnetic moments (*f* spins) through the *s*-*f* exchange interaction. Therefore, the total Hamiltonian $H_{t}$ is expressed as

(1) $$\begin{array}{ccc} H_{t} & = & H_{s}+H_{f}+H_{sf}\; \end{array}$$

where

(2) $$\begin{array}{ccc} H_{s} & = & \sum_{k,\mu }\varepsilon_{k}a_{k\mu }^{†}a_{k\mu } \end{array}$$

(3) $$\begin{array}{ccc} H_{f} & = & -\sum_{mn}J_{mn}S_{m}\cdot S_{n} \end{array}$$

(4) $$\begin{array}{ccc} H_{sf} & = & -I\sum_{n\mu ,\nu }a_{n\mu }^{†}\sigma_{\mu \nu }\cdot S_{n}a_{n\nu }\; \end{array}$$

Here, $H_{s}$ represents the translational energy of an *s* electron; $a_{k\mu }^{†}$ and $a_{k\mu }$ are, respectively, the creation and annihilation operators for the Bloch state of an *s* electron with spin *μ* and wave vector *k*, and $\varepsilon_{k}$ is the energy of the Bloch state. $H_{f}$ is the Heisenberg-type exchange interaction between *f* spins; $S_{n}$ is the operator of the *f* spin located at the *n*th lattice site, and $J_{mn}$ is the exchange interaction constant between *f* spins at the *m*th and *n*th sites. The *s*-*f* exchange interaction between an *s* electron and *f* spins, $H_{sf}$, is represented by the simplest form of the intra-atomic exchange model; *I* is the exchange constant and $\sigma_{\mu \nu }$ is an element of the Pauli matrix for an *s* electron; $a_{n\mu }^{†}$ and $a_{n\mu }$ are the creation and annihilation operators for the Wannier states of an *s* electron with spin *μ* at site *n*, respectively.

Generally, magnetic excitation energy is very small compared with the conduction bandwidth and the *s*-*f* exchange energy. Thus, we treat the *f* spins as a quasi-static system, that is, we take the thermal average for fluctuating *f* spins at the final stage of the derivation of physical quantities. Throughout this paper, we shall confine our discussion to the so-called one-particle picture. Thus, we define the single-particle Green’s function as

(5) $$\begin{array}{ccc} G(\omega ) & = & \frac{1}{\omega -H}\; \end{array}$$

with

(6) $$\begin{array}{ccc} H & = & H_{s}+H_{sf}\; \end{array}$$

and write its thermal average for *f* spins as 〈G(ω)〉. Hereafter, the variable *ω* will be omitted from the operators for cases where this will cause no confusion.

To apply the multiple-scattering theory [28,29], we divide *H* into the unperturbed Hamiltonian *K* and the perturbation term *V*. When magnetization arises, an *s* electron in an FMS is subjected to different effective potentials through the *s*-*f* exchange interaction depending on the orientation of its spin. Thus, we assume a spin-dependent effective medium in which a carrier is subject to a coherent potential, $\Sigma_{↑}$ or $\Sigma_{↓}$, according to the orientation of its spin. The coherent potential $\Sigma_{↑}$ ($\Sigma_{↓}$) is an energy (*ω*)-dependent complex potential. Then, an *s* electron moving in this effective medium is described by the (unperturbed) reference Hamiltonian *K*:

(7) $$\begin{array}{ccc} K & = & \sum_{k\mu }\left(\varepsilon_{k}+\Sigma_{\mu }\right)a_{k\mu }^{†}a_{k\mu }\; \end{array}$$

Thus, the perturbation term V(≡H−K) is written as the following sum over each lattice site:

(8) $$\begin{array}{ccc} V & = & \sum_{n}v_{n}\;\; \end{array}$$

with

(9) $$\begin{array}{ccc} v_{n} & = & \sum_{\mu ,\nu }a_{n\mu }^{†}\left(-I\sigma_{\mu \nu }\cdot S_{n}-\Sigma_{\mu }\delta_{\mu \nu }\right)a_{n\mu } \end{array}$$

Next, using the reference Green’s function *P* given by

(10) $$\begin{array}{ccc} P(\omega ) & = & \frac{1}{\omega -K}\;\; \end{array}$$

we define the *t*-matrix of the *s*-*f* exchange interaction due to a localized spin $S_{n}$ embedded in the effective medium by

(11) $$\begin{array}{ccc} t_{n} & = & v_{n}{\left[1-Pv_{n}\right]}^{-1}\; \end{array}$$

Note that $t_{n}$ represents the complete scattering associated with the isolated potential $v_{n}$ in the effective medium, and that *K*, and thus *P*, includes no localized spin operator. According to the multiple-scattering theory, the total scattering operator *T*, which is related to G≡1/(ω−H) as

(12) $$\begin{array}{ccc} G & = & P+PTP\; \end{array}$$

is expressed as the multiple-scattering series

(13) $$\begin{array}{ccc} T & = & \sum_{n}t_{n}+\sum_{n}t_{n}P\sum_{m\;(\neq n)}t_{m}+\sum_{n}t_{n}P\sum_{m\;(\neq n)}t_{m}P\sum_{l\;(\neq m)}t_{l}+\cdots \; \end{array}$$

Within the single-site approximation, the condition

(14) $$\begin{array}{ccc} \langle t_{n}\rangle & = & 0,\;\mathrm{for}\;\mathrm{any}\;\mathrm{site}\;n\; \end{array}$$

leads to 〈T〉≅0 , and thus 〈G〉≅P. This is the CPA.

Here, we introduce the diagonal component of *P* in the Wannier representation, $F_{\mu }\left(\omega \right)\equiv \langle n\mu |P\left(\omega \right)|n\mu \rangle$ (independent of *n*), as

(15) $$\begin{array}{ccc} F_{\mu }\left(\omega \right) & \equiv & \langle n\mu |P\left(\omega \right)|n\mu \rangle =\frac{1}{N}\sum_{k}\frac{1}{\omega -\varepsilon_{k}-\Sigma_{\mu }}\; \end{array}$$

In the CPA, the coherent potential $\Sigma_{\mu }$ is set to satisfy the condition Equation (14) so that 〈G〉≅P. Thus, the density of states (DOS) of the electron with spin μ(=↑,or↓) is calculated as

(16a) Dμ(ω)=1NTr〈μ|〈δ(ω−H)〉|μ〉=−1NπImTr〈μ|〈1ω+i0−H〉|μ〉

(16b) ≅−1NπImTr〈μ|1ω−K|μ〉=−1NπIm∑k1ω−εk−Σμ=−1πImFμ(ω)

### 2.2. t-Matrix Elements and Their Thermal Average

Here we show that the *t*-matrix elements of the *s*-*f* exchange interaction for the *f* spin embedded in the effective medium, $\Sigma_{↑}$ ($\Sigma_{↓}$), can be derived without further approximation [33,43]. Because the exchange term *v* has four spin-matrix elements, the *t*-matrix also has four components. For simplicity, we will omit the site suffix *n* in the Wannier representation; $t_{\mu \nu }\equiv \langle n\mu |t|n\nu \rangle$, where μ,ν=↑ and/or ↓. In accordance with the definition of the *t*-matrix in Equation (11), we have

(17) $$\begin{array}{ccc} t[1-Pv] & = & v\; \end{array}$$

Equation (17) is written in the spin-matrix-element expression as

(18a) $$\begin{array}{ccc} t_{↑↑}-t_{↑↑}F_{↑}v_{↑↑}-t_{↑↓}F_{↓}v_{↓↑} & = & v_{↑↑}\; \end{array}$$

(18b) $$\begin{array}{ccc} t_{↑↓}-t_{↑↓}F_{↓}v_{↓↓}-t_{↑↑}F_{↑}v_{↑↓} & = & v_{↑↓}\; \end{array}$$

where $F_{\mu }\equiv \langle \mu |P|\mu \rangle$. Then, Equation (18a) ×${\left(F_{↓}v_{↓↑}\right)}^{-1}$ + Equation (18b) × ${\left(1-F_{↓}v_{↓↓}\right)}^{-1}$ leads to an equation including $t_{↑↑}$ only ($t_{↑↓}$ is canceled):

(19) $$\begin{array}{c} \;t_{↑↑}\left[\left(1-F_{↑}v_{↑↑}\right){\left(F_{↓}v_{↓↑}\right)}^{-1}-F_{↑}v_{↑↓}{\left(1-F_{↓}v_{↓↓}\right)}^{-1}\right]=v_{↑↑}{\left(F_{↓}v_{↓↑}\right)}^{-1}+v_{↑↓}{\left(1-F_{↓}v_{↓↓}\right)}^{-1}\; \end{array}$$

Recalling the commutation relationships between the components of **S**,

(20) $$\begin{array}{ccc} S_{-}S_{z} & = & \left(S_{z}+1\right)S_{-}\; \end{array}$$

(21) $$\begin{array}{ccc} {\left(S_{+}\right)}^{-1} & = & {\left[S\left(S+1\right)-{\left(S_{z}\right)}^{2}-S_{z}\right]}^{-1}\left(S_{-}\right)\; \end{array}$$

we obtain an explicit expression for $t_{↑↑}$ using no further approximations. Here, *S* is the magnitude of the localized spin S, and the *z* component of the *f* spin is $S_{z}$: $S_{+}=S_{x}+iS_{y}$ and $S_{-}=S_{x}-iS_{y}$. Other *t*-matrix elements are also obtained by a similar procedure.

In order to show the resulting expressions simply, it is convenient to introduce the following symbols:

(22a) $$\begin{array}{ccc} V_{↑} & \equiv & v_{↑↑}=-IS_{z}-\Sigma_{↑}\; \end{array}$$

(22b) $$\begin{array}{ccc} V_{↓} & \equiv & v_{↓↓}=+IS_{z}-\Sigma_{↓}\; \end{array}$$

(22c) $$\begin{array}{ccc} v_{↑↓} & = & -IS_{-} \end{array}$$

(22d) $$\begin{array}{ccc} v_{↓↑} & = & -IS_{+} \end{array}$$

(22e) $$\begin{array}{ccc} U_{↑} & \equiv & -I\left(S_{z}-1\right)-\Sigma_{↑}\; \end{array}$$

(22f) $$\begin{array}{ccc} U_{↓} & \equiv & +I\left(S_{z}+1\right)-\Sigma_{↓}\; \end{array}$$

(22g) $$\begin{array}{ccc} W_{↑} & \equiv & I^{2}S_{-}S_{+}=I^{2}\left[S\left(S+1\right)-S_{z}^{2}-S_{z}\right]\; \end{array}$$

(22h) $$\begin{array}{ccc} W_{↓} & \equiv & I^{2}S_{+}S_{-}=I^{2}\left[S\left(S+1\right)-S_{z}^{2}+S_{z}\right]\; \end{array}$$

The physical meanings of the above symbols can be easily explained. $V_{↑}$ $\left(V_{↓}\right)$ is the spin-diagonal component of the *s*-*f* exchange interaction, wherein an *s* electron with ↑(↓) spin interacts with an *f* spin embedded in the medium of $\Sigma_{↑}$ ($\Sigma_{↓}$). $U_{↑}$ $\left(U_{↓}\right)$ is the spin-diagonal component of the *s*-*f* exchange interaction, wherein an *s* electron with ↑(↓) spin interacts with an *f* spin that has already flipped in the previous scattering; thus, the *f* spin operator $S_{z}$ is replaced by $S_{z}-1$ ($S_{z}+1$). Both $V_{↑}$ $\left(V_{↓}\right)$ and $U_{↑}$ $\left(U_{↓}\right)$ describe the scattering process without a spin flip. On the other hand, $W_{↑}$ ($W_{↓}$) represents the interaction energy required by an *s* electron with ↑ (↓) spin to flip and then reverse its spin after intermediate propagation with a flipped spin. The resulting expressions are

(23a) $$\begin{array}{ccc} t_{↑↑} & = & \frac{V_{↑}+F_{↓}\left(W_{↑}-V_{↑}U_{↓}\right)}{1-F_{↓}U_{↓}-F_{↑}\left[V_{↑}+F_{↓}\left(W_{↑}-V_{↑}U_{↓}\right)\right]}\; \end{array}$$

(23b) $$\begin{array}{ccc} t_{↓↓} & = & \frac{V_{↓}+F_{↑}\left(W_{↓}-V_{↓}U_{↑}\right)}{1-F_{↑}U_{↑}-F_{↓}\left[V_{↓}+F_{↑}\left(W_{↓}-V_{↓}U_{↑}\right)\right]}\; \end{array}$$

(23c) $$\begin{array}{ccc} t_{↑↓} & = & \frac{1}{1-F_{↓}U_{↓}-F_{↑}\left[V_{↑}+F_{↓}\left(W_{↑}-V_{↑}U_{↓}\right)\right]}\left(-IS_{-}\right)\;\\ & = & \left(-IS_{-}\right)\frac{1}{1-F_{↑}U_{↑}-F_{↓}\left[V_{↓}+F_{↑}\left(W_{↓}-V_{↓}U_{↑}\right)\right]}\; \end{array}$$

(23d) $$\begin{array}{ccc} t_{↓↑} & = & \frac{1}{1-F_{↑}U_{↑}-F_{↓}\left[V_{↓}+F_{↑}\left(W_{↓}-V_{↓}U_{↑}\right)\right]}\left(-IS_{+}\right)\;\\ & = & \left(-IS_{+}\right)\frac{1}{1-F_{↓}U_{↓}-F_{↑}\left[V_{↑}+F_{↓}\left(W_{↑}-V_{↑}U_{↓}\right)\right]}\; \end{array}$$

Note that the diagonal-matrix element $t_{\mu \mu }$ involves only $S_{z}$ as an operator [*i.e.*, $t_{\mu \mu }\equiv t_{\mu \mu }\left(S_{z}\right)$]. Thus, the thermal average over the fluctuation of the localized spin is taken as

(24) $$\langle t_{\mu \mu }\rangle =\sum_{S_{z}=-S}^{S}t_{\mu \mu }\left(S_{z}\right)\exp\left(\frac{hS_{z}}{k_{B}T}\right)/\sum_{S_{z}=-S}^{S}\exp\left(\frac{hS_{z}}{k_{B}T}\right)\;$$

where *h* denotes the effective field acting on the localized *f* spins. Since there is a one-to-one correspondence between $\langle S_{z}\rangle$ and the parameter $\lambda \equiv h/k_{B}T$ through the relationship

(25) $$\langle Sz\rangle =\sum_{S_{z}=-S}^{S}S_{z}\exp\left(\frac{hS_{z}}{k_{B}T}\right)/\sum_{S_{z}=-S}^{S}\exp\left(\frac{hS_{z}}{k_{B}T}\right)\;$$

we can describe the electron states in terms of $\langle S_{z}\rangle$ instead of *λ*. Note that the thermal average of the off-diagonal elements $\langle t_{↑↓}\rangle =\langle t_{↓↑}\rangle$ is 0 because the magnetization is assumed to be along the *z* axis.

The conditions for the CPA are expressed as

(26a) $$\begin{array}{ccc} \langle t_{↑↑}\rangle & = & 0\; \end{array}$$

(26b) $$\begin{array}{ccc} \langle t_{↓↓}\rangle & = & 0\; \end{array}$$

It is worth noting that the spin-flip processes are properly taken into account in the above expression for the *t*-matrix elements. As a result, each *t* matrix element $t_{\mu \mu }$ depends on both $\Sigma_{↑}$ and $\Sigma_{↓}$ Equations (26a) and (26b) simultaneously.

For an undisturbed DOS, we assume the model DOS to have a semicircular form with a half-bandwidth Δ:

(27) $$\begin{array}{ccc} \rho (\varepsilon ) & = & \frac{2}{\pi \Delta }\sqrt{1-{\left(\frac{\varepsilon }{\Delta }\right)}^{2}}\; \end{array}$$

Then, $F_{\mu }\left(\omega \right)$ is calculated as

(28) $$\begin{array}{ccc} F_{\mu }\left(\omega \right) & = & \frac{1}{N}\sum_{k}\frac{1}{\omega -\varepsilon_{k}-\Sigma_{\mu }}=\int_{-\Delta }^{\Delta }\frac{\rho (\varepsilon )}{\omega -\varepsilon -\Sigma_{\mu }}d\varepsilon \end{array}$$

(29) $$\begin{array}{ccc} & = & \frac{2}{\Delta }\left\{\left(\frac{\omega -\Sigma_{\mu }}{\Delta }\right)-\sqrt{{\left(\frac{\omega -\Sigma_{\mu }}{\Delta }\right)}^{2}-1}\right\}\; \end{array}$$

The procedure of the numerical calculation is as follows. For a given *ω*, by assigning a suitable complex value of $\Sigma_{\mu }$ (for μ=↑ or ↓), $F_{\mu }$ is calculated by Equation (29). Taking the thermal average for fluctuating *f* spins using Equation (24), $\Sigma_{↑}$ and $\Sigma_{↓}$ are simultaneously determined by Equation (26). Then, we can calculate $F_{\mu }$ again. This procedure is repeated until the calculation converges (see Reference [33] for details). In all of the present numerical results, we have numerically verified that

(30) $$\begin{array}{c} \int_{-\infty }^{\infty }D_{↑}\left(\omega \right)d\omega =\int_{-\infty }^{\infty }D_{↓}\left(\omega \right)d\omega =1 \end{array}$$

### 2.3. Results for the Conduction-electron States in an FMS

The parameters that are necessary to describe the present model are the conduction bandwidth 2Δ, the exchange energy IS(=I×S), the magnetization $\langle S_{z}\rangle /S$, and the quantum spin factor 1/S. Here, we take S=7/2 for the *f* spin. We first confirm that the exchange interaction term −Iσ·S has two eigenstates (*i.e.*, the parallel-coupling state and antiparallel-coupling state) according to the manner of coupling between the *s* electron’s spin and the localized *f* spin. The parallel-coupling state (denoted by p) has an energy eigenvalue of $\varepsilon_{p}=-IS$ with the degeneracy of 2S+2, while the antiparallel-coupling state (denoted by a) has an energy eigenvalue of $\varepsilon_{a}=+I\left(S+1\right)$ with the degeneracy of 2S. Therefore, the band splits into two subbands when the exchange energy IS is large compared with the bandwidth 2Δ.

**Figure 1:**
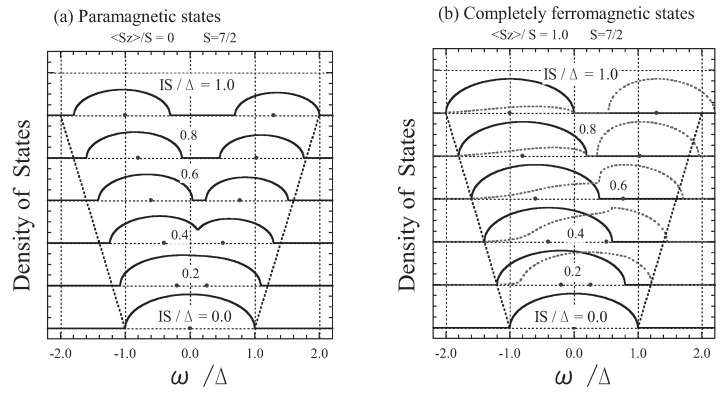
The DOS shown as a function of *ω*/Δ for IS/Δ = 0.0, 0.2, 0.4, 0.6, 0.8, and 1.0: (a) paramagnetic states ($\langle S_{z}\rangle$ = 0; left panel) and (b) completely ferromagnetic states ($\langle S_{z}\rangle$ = S; right panel). The solid curves show up-spin states and the dotted curves show down-spin states. The points on the energy axes indicate −IS and +I(S+1), and the straight dotted lines indicate −(Δ+IS) and +(Δ+IS) for reference.

In Figure 1(a), we show the DOS for various exchange energies IS/Δ in the paramagnetic states. At high temperatures the orientation of *f* spins is completely random. Therefore, the present result is equivalent to that obtained by Rangette etal. [31], who applied the CPA to an electron propagating in a disordered binary alloy in which two kinds of atoms with atomic energies of −IS and +I(S+1) are randomly distributed with concentrations of (S+1)/(2S+1) and S/(2S+1), respectively. When IS/Δ is small, the band remains as a single band, while it broadens with the increase of IS/Δ. For I(2S+1)≳Δ (or IS/Δ≳0.44) the band splits into two subbands, the parallel-coupling and antiparallel-coupling subbands, whose centers are at −IS and +I(S+1), and whose total numbers of states are 2(S+1)/(2S+1)=1.125 and 2S/(2S+1)=0.875, respectively.

In Figure 1(b), we show the DOS in the completely ferromagnetic states. In this case, only the value $S_{z}=S$ is realized in the thermal average over the *f* spin states. Hence, from Equation (26) we obtain

(31a) $$\begin{array}{ccc} \Sigma_{↑} & = & -IS\; \end{array}$$

(31b) $$\begin{array}{ccc} \Sigma_{↓} & = & +IS\frac{\left(1+IF_{↑}\right)}{\left(1-IF_{↑}\right)}\; \end{array}$$

The results can be interpreted as follows. For the completely ferromagnetic case (*i.e.*, T=0), the states of an *s* electron with up spin only shift by −IS with no damping. On the other hand, the *s*-electron states with down spin are damped because they can flip their spin under the condition that the total spin (=S−1/2) is conserved if the DOS with up spin is not zero therein. This is because $\Sigma_{↓}$ involves $F_{↑}\;\left[\equiv F_{↑}\left(\omega \right)\right]$. This spin-flip process of the *s* electron is a quantum effect due to the finiteness of the magnitude of the *f* spin. Thus, in the classical spin limit [*i.e.*, setting S≫1 and $S_{z}\gg 1$ in Equation (23)] , Equation (31b) is replaced by

(32) $$\begin{array}{ccc} \Sigma_{↓} & = & -IS \end{array}$$

**Figure 2:**
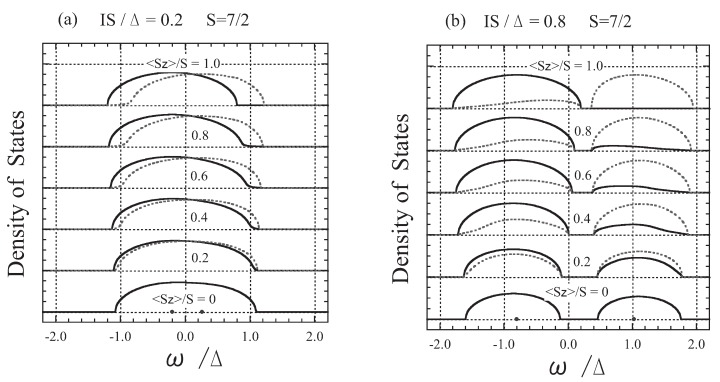
The DOS shown for magnetizations $\langle S_{z}\rangle /S$ = 0.0, 0.2, 0.4, 0.6, 0.8, and 1.0: (a) IS/Δ=0.2 (left panel) and (b) IS/Δ=0.8 (right panel). The solid curves show up-spin states and the dotted curves show down-spin states.

In Figure 2, we show the DOS for two typical cases: (a) weak exchange interaction (left panel; IS/Δ=0.2) and (b) strong exchange interaction (right panel; IS/Δ=0.8). In the weak exchange interaction limit (IS/Δ≃0), the present results agree with those obtained using first-order perturbation theory. Substituting Equation (23a) into Equation (26a) and Equation (23b) into Equation (26b), and expanding them in *I* to the first order, we obtain

(33a) $$\begin{array}{ccc} \langle V_{↑}\rangle & = & 0\;\text{then}\;\Sigma_{↑}=-I\langle S_{z}\rangle \; \end{array}$$

(33b) $$\begin{array}{ccc} \langle V_{↓}\rangle & = & 0\;\text{then}\;\Sigma_{↓}=+I\langle S_{z}\rangle \; \end{array}$$

This means that the ferromagnetic ordering of *f* spins gives rise to the $-I\langle S_{z}\rangle$ shift in the up-spin band and the $+I\langle S_{z}\rangle$ shift in the down-spin band. However, even when IS/Δ=0.2, this is not the case, as is shown in Figure 2(a). The bands are broadened owing to the fluctuation of the *f* spins, and the down-spin band has a tail which reaches the bottom of the up-spin band even in the case of completely ferromagnetic states ($\langle S_{z}\rangle =S$). This explains why the electron-spin polarization cannot reach 100%; the origin is the quantum effect coming from the finiteness of the magnitude of the *f* spin, as already discussed [34].

As shown in Figure 2(b), in a case of the strong exchange interaction, the band splits into two subbands depending on the coupling of the *s* electron spin and *f* spins: the parallel-coupling subband (lower-energy side) and the antiparallel-coupling subband (higher-energy side). The total number of states in the parallel-coupling subband per site is 2(S+1)/(2S+1)=1.125 and that in the antiparallel-coupling subband is 2S/(2S+1)=0.875, irrespective of the value of $\langle S_{z}\rangle$. When $\langle S_{z}\rangle =0$, both subbands are composed of the same number of up- and down-spin states. When $\langle S_{z}\rangle =S$, on the contrary, all states in the antiparallel-coupling subband have down spin, while the states in the parallel-coupling subband are composed of all the up-spin states and part of the down-spin states; the number of states with down spin per site is 0.125 in the parallel-coupling subband.

**Figure 3:**
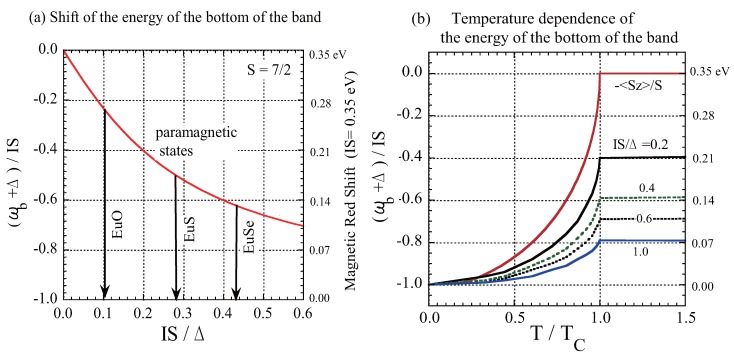
(a) The shift of the bottom of the band in paramagnetic states normalized by the exchange energy, $(\omega_{b}+\Delta )/IS$, shown as a function of IS/Δ. The arrows indicate the experimentally obtained magnetic redshifts of EuO (0.27 eV), EuS (0.18 eV), and EuSe (0.13 eV) with IS=0.35 eV. (b) The energy of the bottom of the band depicted as a function of $T/T_{c}$ for IS/Δ=0.2,0.4,0.6, and 1.0. The value of $-\langle S_{z}/S\rangle$, which corresponds to the result of the weak exchange interaction limit, is also shown.

The energy of the bottom of the band $\omega_{b}$ is shifted by the *s*-*f* exchange interaction from that of the undisturbed (model) band (ω=−Δ), even when $\langle S_{z}\rangle =0$. In Figure 3(a), we show the energy shift of the bottom of the band in the paramagnetic states normalized by IS, $(\omega_{b}+\Delta )/IS$, as a function of IS/Δ; the exact solution for $\omega_{b}$ is given in the Appendix of Reference [38]. Using the result in Figure 3(a), we can explain why Eu chalcogenide FMSs exhibit different redshifts despite the fact that they all have the same lattice structure, the same *f* spin value of S=7/2, and almost the same exchange interaction energy IS [47]. Since the optical absorption band is assigned to the $4f^{7}\to 4f^{6}5d\;\left(t_{2g}\right)$ band transition, the redshift is ascribed to the lowering of the energy of the bottom of the (*d*-like) conduction band with the decrease in temperature due to the (*d*-*f*) exchange interaction [1]. In Figure 3(a), we indicate the magnetic redshifts experimentally observed for EuO (0.27 eV), EuS (0.18 eV), and EuSe (0.13 eV) with arrows. In this approach, $\omega_{b}$ is a function of IS/Δ at paramagnetic temperatures, while $\omega_{b}=-IS$ (independent of Δ) at T=0. Since the exchange interaction has an intra-atomic character due to the strong localization of *f* electrons within the Eu${}^{2+}$ ion, the value of IS does not greatly differ among these Eu chalcogenides. Thus, the difference in the total redshift can be ascribed to the difference in the bandwidth 2Δ. From Figure 3(a), using IS=0.35 eV we estimated the values of the bandwidth (2Δ) as 7 eV (EuO), 2.5 eV (EuS), and 1.6 eV (EuSe). Though uncertainty remains due to the experimental error in measuring the position of the absorption edge, the broad conduction-band picture for EuO is consistent with the result recently obtained by spin-resolved spectroscopy [12]. The tendency that the width of the conduction band of Eu*X* decreases with the change of chalcogenides from X= O to Se is consistent with the electronic structure obtained by optical measurement [1].

In Figure 3(b), the energy shift of the bottom of the band is shown as a function of normalized temperature $T/T_{c}$. From Figure 3(b), we can also explain the reason for the apparent success of first-order perturbation theory in explaining the magnetic redshift. The temperature dependence of the shift in the energy of the bottom of the band is approximately $-I_{\mathrm{eff}}\langle S_{z}\rangle$. Thus, $I_{\mathrm{eff}}$ can be regarded to be the effective exchange constant in which the effect of multiple scattering has already been renormalized.

The results obtained by applying the dynamical CPA for the *s*-*f* model of FMSs are consistent with the experimental observation of the electron-spin polarization [34], and they explain the temperature dependence of the electrical resistivity of degenerate FMSs [35]. We should also add that the coherent potential approach can explain not only the redshift of FMSs but also the blue shift of the antiferromagnetic semiconductor EuTe [36,37].

## 3. Carrier States in Diluted Magnetic Semiconductors (DMSs)

### 3.1. Model Hamiltonian for a Carrier in a DMS and the Application of the Dynamical CPA

In order to study the effect of the *sp-d* exchange interaction between a carrier (an *s* conduction electron or *p* hole) and localized magnetic moments (*d* spins) together with magnetic and chemical disorder in DMSs, we introduce a simple model for $A_{1-x}$Mn${}_{x}B$-type DMSs. In this model, the local potentials of nonmagnetic (*A*) ions in a semiconducting compound (AB) are substituted randomly, with mole fraction (x), by local potentials that include the exchange interaction between a carrier and the localized spin moment on a Mn (denoted by *M*) ion. Thus, the potential to which a carrier is subjected at a site depends on whether the site is occupied by an *A* ion or *M* ion. The Hamiltonian *H* is given by

(34) $$\begin{array}{ccc} H & = & \sum_{m,n,\mu }\varepsilon_{mn}a_{m\mu }^{†}a_{n\mu }\;+\sum_{n}u_{n} \end{array}$$

where $u_{n}$ is either $u_{n}^{A}$ (at the *A* site) or $u_{n}^{M}$ (at the *M* site), depending on the ion species occupying the *n* site:

(35) $$\begin{array}{ccc} u_{n}^{A} & = & E_{A}\sum_{\mu }a_{n\mu }^{†}a_{n\mu }\; \end{array}$$

(36) $$\begin{array}{ccc} u_{n}^{M} & = & E_{M}\sum_{\mu }a_{n\mu }^{†}a_{n\mu }-I\sum_{\mu ,\nu }a_{n\mu }^{†}\sigma_{\mu \nu }\cdot S_{n}a_{n\nu }\; \end{array}$$

Here, $a_{n\mu }^{†}$ and $a_{n\mu }$ are, respectively, the creation and annihilation operators for a carrier with spin *μ* at the *n* site. The transfer-matrix element between *m* and *n*, $\varepsilon_{mn}$, is assumed to be independent of the types of constituent atoms that occupy the *m* and *n* sites. In II-VI-based DMSs of the $A_{1-x}^{\mathrm{II}}{\mathrm{Mn}}_{x}B^{\mathrm{VI}}$-type, $E_{A}$ ($E_{M}$) represents a nonmagnetic local potential at the $A^{2+}$ (Mn${}^{2+}$) sites. In III-V-based DMSs such as Ga${}_{1-x}$Mn${}_{x}$As, the spin-independent potential $E_{M}\left(<0\right)$ can be regarded as a screened Coulomb attractive potential between a carrier (hole) and the Mn${}^{2+}$ ion (acceptor center). The exchange interaction between the carrier and localized spin $S_{n}$ of the Mn site *n* is expressed by $-Ia_{n\mu }^{†}\sigma_{\mu \nu }\cdot S_{n}a_{n\nu }$, where $\sigma_{\mu \nu }$ represents the element of the Pauli spin matrices. We disregard the electron-electron, hole-hole, and/or electron-hole interactions.

The application of the dynamical CPA to the Hamiltonian in Equation (34) is straightforward [41,42,43]. Since the present system includes both substitutional disorder and the thermal fluctuation of the localized spin at an *M* site, the average of the *t*-matrix is written as

(37) $$\begin{array}{ccc} {\langle t\rangle }_{\mathrm{av}} & = & \left(1-x\right)t^{A}+x\langle t^{M}\rangle \; \end{array}$$

Here, we express the average of $t_{n}$ over the disorder in the system as ${\langle t_{n}\rangle }_{\mathrm{av}}$; (1−x) and *x* are the mole fractions of *A* and *M* atoms, respectively. $t^{A}$ is the *t*-matrix that represents the multiple scattering of a carrier due to the *A* ion potential $u^{A}$ [in Equation (35)] embedded in the effective medium. Assuming the spin-dependent coherent potential $\Sigma_{\mu }$ (μ=↑ or ↓), the *t*-matrix elements are given as [29]

(38) $$\begin{array}{ccc} t_{\mu \mu }^{A} & = & \frac{E_{A}-\Sigma_{\mu }}{1-\left(E_{A}-\Sigma_{\mu }\right)F_{\mu }}\; \end{array}$$

Here, $F_{\mu }$ is a diagonal matrix element of a propagator with respect to the effective medium and is given by Equations (15) and/or (29). $t^{M}$ is the *t*-matrix that represents the multiple scattering of a carrier due to the *M* ion potential $u^{M}$ [in Equation (36)] embedded in the effective medium; $\langle t^{M}\rangle$ is the thermal average of $t^{M}$ over the fluctuation of the localized spin. Explicit expressions for $t_{\mu \nu }$ are obtained after minor substitutions in Equation (23); $\Sigma_{\mu }\to \Sigma_{\mu }-E_{M}$. In the dynamical CPA, the coherent potential $\Sigma_{\mu }$ is set such that the effective scattering of a carrier at the chosen site embedded in the effective medium is zero on average. Thus, the dynamical CPA conditions are given by

(39a) $$\begin{array}{ccc} \left(1-x\right)t_{↑↑}^{A}+x\langle t_{↑↑}^{M}\rangle & = & 0\; \end{array}$$

(39b) $$\begin{array}{ccc} \left(1-x\right)t_{↓↓}^{A}+x\langle t_{↓↓}^{M}\rangle & = & 0\; \end{array}$$

In Appendix A, we outline the dynamical CPA using locator formalism, which has been proved to be equivalent with the dynamical CPA using *t*-matrix formalism. The advantage of the locator formalism is that it can be easily used to obtain the species-resolved DOSs $D_{\mu }^{A}\left(\omega \right)$ and $D_{\mu }^{M}\left(\omega \right)$, which are the DOSs with *μ* spin associated with an *A* ion and *M* ion, respectively.

### 3.2. General Consideration for the Carrier States in a DMS

In the subsections below, we treat the localized spins classically, although S=5/2 for the Mn${}^{2+}$ ion. One of the advantages of the classical spin approximation is that it reduces the number of physical parameters. It is sufficient to assign a value to the exchange energy IS=I×S instead of assigning the values of *I* and *S* separately. In the classical spin approximation, we let 1/S approach 0 (*i.e.*, S→∞) while keeping IS constant. The quantum fluctuation of the localized spin is ignored. As a consequence, both eigenstates have the degeneracy of 2S, and the eigenenergies become symmetric: $\varepsilon_{p}=-IS$ and $\varepsilon_{a}=+IS$. The *s-d* exchange interaction between a conduction electron (*s* electron) and a localized spin (*d* spin) favors parallel coupling. On the other hand, in most DMSs, the *p-d* exchange interaction between a hole (*p* hole) and a localized spin favors antiparallel coupling, and the magnitude of the *p*-*d* exchange interaction is several times larger than that of the *s-d* exchange interaction. The *p-d* exchange interaction plays an important role in magneto-optical effects in II-VI-based DMSs and is related to the carrier-induced ferromagnetism in III-V-based DMSs. Hence, keeping the *p-d* exchange interaction in mind, we assume IS<0 hereafter. Note that the present model requires only two parameters, IS/Δ and $E_{M}/\Delta$, after we set $E_{A}\equiv 0$.

In Section 3.3, Section 3.4 and Section 3.5, we will discuss some typical cases of $A_{1-x}M_{x}B$-type DMSs in which 5% of the nonmagnetic (*A*) ions are randomly substituted by magnetic (*M*) ions. The results reveal the nature of the magnetic impurity bands and how the carrier states behave with changing magnetization. In Section 3.6, based on the Curie temperature $T_{c}$ calculated in a simple way, we will discuss the type and properties of ferromagnetism which may occur when carriers introduced into $A_{1-x}M_{x}B$-type DMSs. In Section 3.7, we will focus on the case of (Ga, Mn)As.

### 3.3. The Case of Strong Exchange Interaction

**Figure 4:**
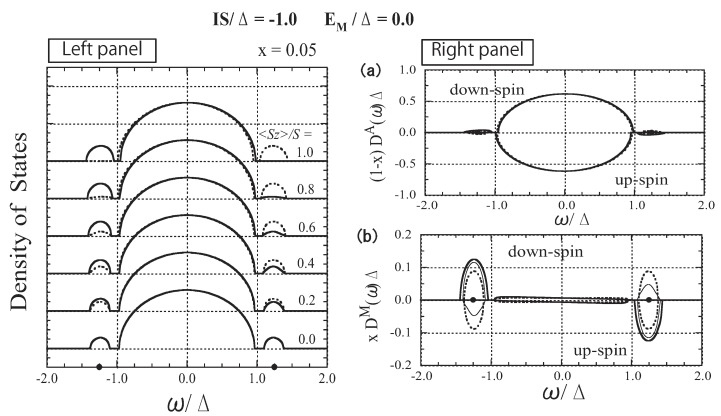
Left panel: DOS as a function of ω/Δ for various values of $\langle S_{z}\rangle /S$. The solid lines represent the down-spin carrier and dotted lines represent the up-spin carrier. The impurity levels $E_{a}=\pm 1.25\Delta$ are indicated by dots on the abscissa. Right panel: (a) *A*-site component of the DOS, $\left(1-x\right)D_{↓}^{A}\left(\omega \right)\Delta$ and $-\left(1-x\right)D_{↑}^{A}\left(\omega \right)\Delta$. (b) *M*-site component of the DOS, $xD_{↓}^{M}\left(\omega \right)\Delta$ and $-xD_{↑}^{M}\left(\omega \right)\Delta$. The thick, thin, and dotted lines represent the cases of $\langle S_{z}\rangle /S=1.0$, 0.5, and 0.0, respectively. Note the difference in the scale of the vertical axes of (a) and (b). From Takahashi and Kubo [42].

In Figure 4, we show the numerical results with IS=−Δ and $E_{M}=0.0$, referred to as the case of strong exchange interaction hereafter. In the left panel of Figure 4, the spin-polarized DOSs, $D_{↑}\left(\omega \right)$ and $D_{↓}\left(\omega \right)$, are depicted for various values of $\langle S_{z}\rangle /S$. In the dilute impurity limit (x→0), impurity levels appear at the energies of $\frac{E_{a}}{\Delta }=\left(\frac{E_{M}\mp IS}{\Delta }\right)+\frac{1}{4}\left(\frac{\Delta }{E_{M}\mp IS}\right)=\pm$1.25. When *x* = 0.05, impurity bands appear around the impurity levels. The total number of states of each impurity band is *x*, irrespective of $\langle S_{z}\rangle$. The lower (higher)-energy impurity band corresponds to the antiparallel (parallel)-coupling state.

The impurity bands are strongly affected by changes in $\langle S_{z}\rangle$. On the other hand, the host band is negligibly affected. To elucidate the origin of the change in the DOS, we calculate the species-resolved DOS. In the left panel of Figure 4, we depict the *A-* and *M*-site components of the DOS, $\left(1-x\right)D_{\mu }^{A}\left(\omega \right)$ and $xD_{\mu }^{M}\left(\omega \right)$, respectively. $D_{\mu }^{A}\left(\omega \right)$ [$D_{\mu }^{M}\left(\omega \right)$] represents the local DOS with *μ* spin (μ=↑ or ↓) associated with the *A* (*M*) ion. Note that

(40) $$\begin{array}{c} D_{\mu }\left(\omega \right)=\left(1-x\right)D_{\mu }^{A}\left(\omega \right)+xD_{\mu }^{M}\left(\omega \right)\; \end{array}$$

Since $D^{A}\left(\omega \right)$ and $D^{M}\left(\omega \right)$ are normalized, the total numbers of *A*-site states and *M*-site states are 1−x and *x*, respectively. The numerical result shown in the right panel reveals that the impurity state is mainly composed of *M*-site states and that the change in the impurity band is mainly ascribed to the change in $D_{\mu }^{M}\left(\omega \right)$.

### 3.4. The Case of Moderate Exchange Strength (II-VI-based DMSs)

**Figure 5:**
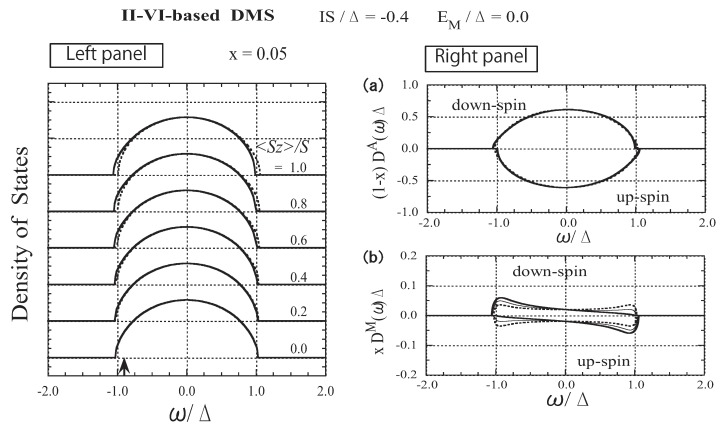
The results for II-VI-based DMSs. Left panel: DOS as a function of ω/Δ for various values of $\langle S_{z}\rangle /S$. The solid lines represent the down-spin carrier and the dotted lines represent the up-spin carrier. The arrow indicates the Fermi level $\varepsilon_{F}/\Delta$ for n=x(=0.05). Right panel: (a) *A*-site component of the DOS, $\left(1-x\right)D_{↓}^{A}\left(\omega \right)\Delta$ and $-\left(1-x\right)D_{↑}^{A}\left(\omega \right)\Delta$. (b) *M*-site component of the DOS, $xD_{↓}^{M}\left(\omega \right)\Delta$ and $-xD_{↑}^{M}\left(\omega \right)\Delta$. The thick, thin, and dotted lines represent the cases of $\langle S_{z}\rangle /S=1.0$, 0.5, and 0.0, respectively. From Takahashi and Kubo [42].

**Figure 6:**
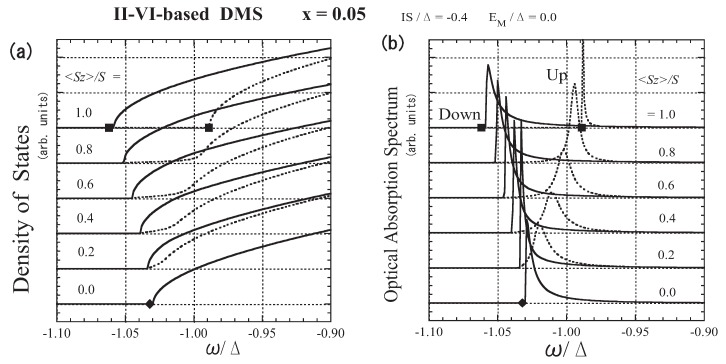
The results for II-VI-based DMSs. (a) Low-energy part of the DOS D(ω). (b) Optical absorption spectrum A(ω) in arbitrary units (arb. units). The solid lines represent the down-spin carrier and the dotted lines represent the up-spin carrier. The values of the band-edge energy, $\omega_{b}/\Delta$, obtained by a simple approximation are plotted as dots on the lines of $\langle S_{z}\rangle =S$ and $\langle S_{z}\rangle =0$ (see text). Note that the energy of the bottom of the model band is ω=−Δ. From Takahashi [43].

**Figure 7:**
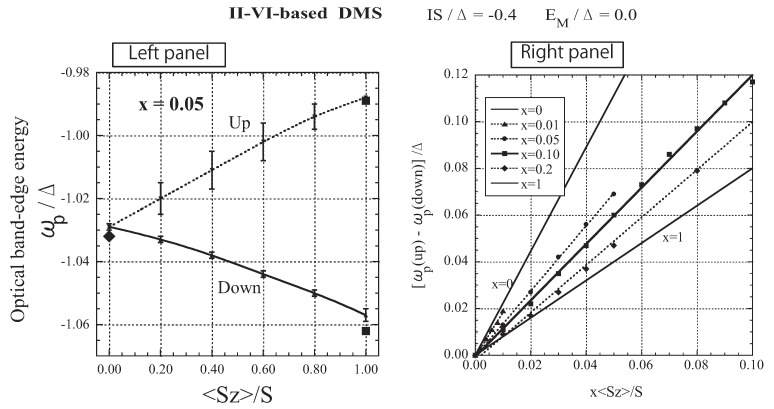
Left panel: Absorption peak energy $\omega_{p}/\Delta$ as a function of $\langle S_{z}\rangle /S$. The solid line represents the down-spin carrier and the dotted line represents the up-spin carrier. The error bar represents the half-peak width. The values of $\omega_{b}/\Delta$ obtained by a simple approximation are plotted as dots on the lines of $\langle S_{z}\rangle =S$ and $\langle S_{z}\rangle =0$ (see text). Right panel: Exchange splitting $[\omega_{p}\left(\mathrm{up}\right)-\omega_{p}\left(\mathrm{down}\right)]/\Delta$ as a function of $x\langle S_{z}\rangle /S$ for various values of *x*. The straight lines are adjusted to obtain the best fit with each set of *x* data. From Takahashi [43].

To the best of our knowledge, no magnetic impurity band has been observed in $A_{1-x}^{\mathrm{II}}{\mathrm{Mn}}_{x}B^{\mathrm{VI}}$-type DMSs [23]. Thus, II-VI-based DMSs correspond to the present model with $|E_{M}\pm IS|<0.5\Delta$. For convenience, we take the exchange energy IS=−0.4Δ and the band offset energy $E_{M}=0.0$ for II-VI-based DMSs. Numerical results for the parameters are presented in Figure 5, Figure 6 and Figure 7. In the left panel of Figure 5, the spin-polarized DOS is shown for various values of $\langle S_{z}\rangle$. As can be seen in Figure 5(b), the carrier states at the *M* site spread over the whole range of band energy, in contrast with the case of strong exchange interaction. The weak $\langle S_{z}\rangle$ dependence of $xD^{M}\left(\omega \right)$ suggests that the coupling between the carrier spin and the localized spin is not strong, except at the band edges.

The most powerful tool for studying the exchange interaction between a carrier and localized spins in II-VI-based DMSs is optical measurement. In most II-VI-based DMSs the energy splitting between $\sigma^{+}$ and $\sigma^{-}$ *A* excitons in a DMS is well described as [23,24]

(41) $$\begin{array}{c} \delta E=N_{0}\left(\alpha -\beta \right)x\langle S_{z}\rangle \end{array}$$

The virtual crystal approximation (VCA) is a first-order perturbation theory with respect to the exchange interaction. When applying the VCA to the present model, we obtain the energy shift in the band edge due to the *M*-site local potential:

(42) $$\begin{array}{ccc} \Sigma_{b}\left(\pm \right) & = & (E_{M}\mp I\langle S_{z}\rangle )x \end{array}$$

depending on the carrier’s spin (±). Therefore, $N_{0}\alpha$ and $N_{0}\beta$ are regarded as the exchange constants for conduction electrons and valence electrons, respectively; $N_{0}\alpha =2I$ for an electron and $N_{0}\beta =2I$ for a hole. However, some experimental results indicate that the application of the VCA is limited [38], although the VCA has been widely accepted as describing the field-induced exchange splitting of extended states in II-VI-based DMSs [23,24,25]. Therefore, it is important to clarify the limit of application of the VCA and to devise a theoretical treatment beyond the VCA.

Here we consider the optical absorption spectrum on the basis of the dynamical CPA treatment in which the multiple-scattering effect is considered [43]. In calculating the optical absorption spectrum, we assume that the transition dipole moments of the *A* and *M* ions are the same. Under this assumption, the optical absorption spectrum is given by the k=0 components of the DOS. Since the explicit *k* dependence of $\varepsilon_{k}$ is not used in the present framework, we assume that k=0 corresponds to the minimum point of the model band. Therefore, taking $\varepsilon_{0}=-\Delta$, we define the optical absorption spectrum as [30]

(43) Aμ(ω)=−1πIm1ω+Δ−Σμ(ω)

Figure 6(a) shows how the band tail is modified and spin-polarized with the development of magnetization. Note that even with $\langle S_{z}\rangle =0$, the band is not the same as the model band, owing to the disorder of the random distribution of *M* ions and the fluctuation of localized spins. With increasing $\langle S_{z}\rangle$, the bottom of the down-spin band shifts to a lower energy, accompanied by an energy shift of the bottom of the up-spin band. The two band edges agree with each other except in the case of $\langle S_{z}\rangle =S$, although the down-spin band is strongly suppressed in the band tail. The agreement of the band edges is a consequence of the spin-flip of a carrier. Thus, the present result for the band-edge shift is very different from that obtained by the VCA. On the other hand, exchange band splitting is observed in magneto-optical measurements such as magneto-absorption and magnetoreflectivity spectra. Hence, we assume that the peak of the optical absorption spectrum A(ω), shown in Figure 6(b), corresponds to the band edge observed in optical measurements. In the right panel of Figure 7, the optical band-edge energy, $\omega_{p}$, at which the optical absorption spectrum shows a peak, is presented for up- and down-spin bands as a function of $\langle S_{z}\rangle$. The behavior of $\omega_{p}$ convincingly explains the asymmetrical splitting of the Zeeman energy component; when a magnetic field is applied, the pattern of spin splitting of the A exciton term is asymmetric relative to the position of $\langle S_{z}\rangle =0$ [40]. In the right panel of Figure 7, the spin-splitting energy $\omega_{p}\left(\text{up}\right)$-$\omega_{p}\left(\text{down}\right)$ is displayed as a function of $x\langle S_{z}\rangle$ for various values of *x*. The data for each *x* are fit by a straight line. With increasing *x*, the slope of the line, corresponding to $N_{0}\beta$, decreases. The same behavior has been experimentally observed in Zn${}_{1-x}$Mn${}_{x}$Te [48] and Cd${}_{1-x}$Mn${}_{x}$Te [49].

Assuming that $\omega_{b}=-\Delta +\Sigma \left(\omega_{b}\right)$ under the dynamical CPA condition [Equation (39)], we can obtain equations for the band-edge energy $\omega_{b}$; the equations are cubic when $\langle S_{z}\rangle =0$ and quadratic when $\langle S_{z}\rangle =S$. The approximate values for the band-edge energy are plotted as dots on the lines of $\langle S_{z}\rangle =S$ and $\langle S_{z}\rangle =0$ in Figure 6(a), (b), and in the left panel of Figure 7. Using the approximate values for the band-edge shift with up and down spins, $\Sigma_{b}\left(+\right)$ and $\Sigma_{b}\left(-\right)$ when $\langle S_{z}\rangle =S$, we calculate $N_{0}\beta$ from $N_{0}\beta =\left[\Sigma_{b}\left(-\right)-\Sigma_{b}\left(+\right)\right]/xS.$ The result shows that $N_{0}\beta$ is a function of *x*, and [39,40]

(44a) $$\begin{array}{ccc} N_{0}\beta & \approx & \frac{2I}{{\left(1+2\frac{E_{M}}{\Delta }\right)}^{2}-{\left(2\frac{IS}{\Delta }\right)}^{2}}\;\mathrm{when}\;x\approx 0 \end{array}$$

(44b) $$\begin{array}{ccc} N_{0}\beta & = & 2I\;\;\mathrm{when}\;x=1\; \end{array}$$

The present treatment reveals that the apparent enhancement of $|N_{0}\beta |$ with decreasing *x* observed in Cd${}_{1-x}$Mn${}_{x}$S is a consequence of the multiple-scattering effect, which is significant for small *x* in a disordered system [39]. The VCA is applicable when $|E_{M}|\ll \Delta$ and |IS|≪Δ [50].

### 3.5. The Case of Strong Attractive Potential

**Figure 8:**
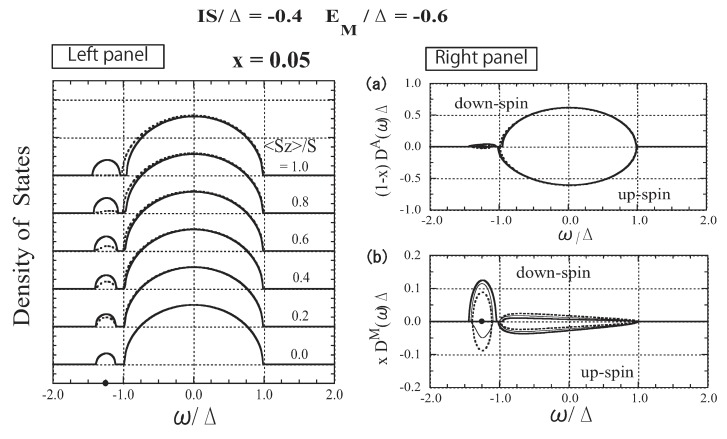
The results for the DMSs with strong attractive potential. Left panel: DOS as a function of ω/Δ for various values of $\langle S_{z}\rangle /S$. The solid lines represent the down-spin carrier and the dotted lines represent the up-spin carrier. The impurity level $E_{a}=-1.25\Delta$ is indicated by the dot on the abscissa. Right panel: (a) *A*-site component of the DOS, $\left(1-x\right)D_{↓}^{A}\left(\omega \right)\Delta$ and $-\left(1-x\right)D_{↑}^{A}\left(\omega \right)\Delta$, (b) *M*-site component of the DOS, $xD_{↓}^{M}\left(\omega \right)\Delta$ and $-xD_{↑}^{M}\left(\omega \right)\Delta$. The thick, thin, and dotted lines represent the cases of $\langle S_{z}\rangle /S=1.0$, 0.5, and 0.0, respectively. From Takahashi and Kubo [42].

Figure 8 shows the results for IS=−0.4Δ and $E_{M}=-0.6\Delta$. Although the same exchange energy, IS=−0.4Δ, as that for the II-VI-based DMSs, is assumed, the nonmagnetic local potential $E_{M}$ generates an impurity band. Furthermore, comparing the DOSs shown in Figure 4 (strong exchange interaction) and Figure 8 (strong attractive potential), it is easy to see a strong similarity. The lower-energy part of the DOS for IS=−0.4Δ and $E_{M}=-0.6\Delta$ is almost the same as that for IS=−Δ and $E_{M}=0.0$. The reason for the similarity can be explained as follows. First, the impurity level is the same, $E_{a}=-1.25\Delta$, because it is determined by the value of $IS+E_{M}\left(=-\Delta \right)$. When $\langle S_{z}\rangle =0$, a magnetic impurity band forms around the impurity level; the number of down- and up-spin states is x/2 each. The impurity band forms in imitation of the model band. When $\langle S_{z}\rangle =S$, the down-spin bands in the two cases agree with each other because the DOSs were calculated for the same value of $E_{M}+IS$. When $\langle S_{z}\rangle =S$, the up-spin bands shift toward higher energies and merge into the host band in both cases, although the up-spin bands do not coincide because different values of $E_{M}-IS$ were used. Therefore, even in the up-spin bands, we observe a similar tendency in the behavior of the two cases. The above similarities of the carrier state in the two cases can be explained as follows. In DMSs with strong attractive potential, the carrier is so strongly attracted to the *M* site due to $E_{M}\left(<0\right)$ that the exchange interaction operates effectively in comparison with the case of $E_{M}=0$. Consequently, the exchange interaction in the case of strong attractive potential yields a very similar effect to that in DMSs with a strong exchange interaction.

### 3.6. Mechanism of Carrier-induced Ferromagnetism in DMSs

In order to study the mechanism of carrier-induced ferromagnetism that may occur when carriers are introduced into DMSs, we calculate the Curie temperature $T_{c}$ in a very simple way. Throughout this article we assume that the carriers are degenerate. Then we obtain the carrier density with *μ* spin $n_{\mu }$ and the total energy $E(\langle S_{z}\rangle )$ by

(45) $$\begin{array}{ccc} n_{\mu } & = & \int_{-\infty }^{\varepsilon_{F}}D_{\mu }\left(\omega \right)d\omega \; \end{array}$$

and

(46) $$\begin{array}{ccc} E(\langle S_{z}\rangle ) & = & \int_{-\infty }^{\varepsilon_{F}}\omega \left[D_{↑}\left(\omega \right)+D_{↓}\left(\omega \right)\right]d\omega \; \end{array}$$

respectively, as functions of the Fermi level $\varepsilon_{F}$. Note that $E(\langle S_{z}\rangle )$ is the sum of the kinetic and exchange energies. For a fixed value of $\langle S_{z}\rangle /S$, the total carrier density $n\;(\equiv n_{↑}+n_{↓})$ has a one-to-one correspondence with $\varepsilon_{F}$ and therefore $E(\langle S_{z}\rangle )$ can be expressed as a function of *n*. Thus, we can estimate $T_{c}$ as a function of *n* using

(47) $$\begin{array}{ccc} k_{B}T_{c} & = & \frac{2}{3x}\;\left[E\left(0\right)-E\left(S\right)\right]\; \end{array}$$

where E(0) and E(S) are the energies of the paramagnetic state and the completely ferromagnetic state, respectively.

**Figure 9:**
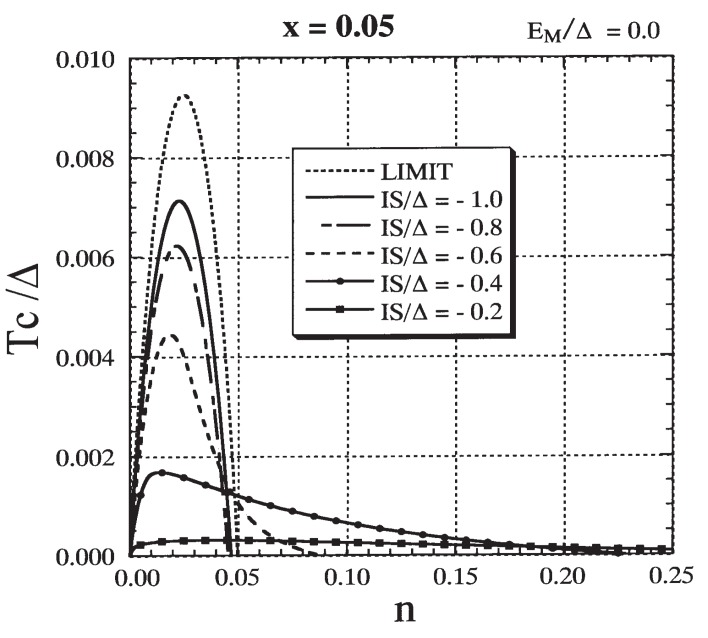
The result for Curie temperature $T_{c}/\Delta$ as a function of carrier density *n* for various values of IS/Δ with x=0.05 and $E_{M}=0$. The result based on the assumption that an impurity band has the same shape as the model DOS is drawn as ‘LIMIT’ (see text). From Takahashi and Kubo [42].

**Figure 10:**
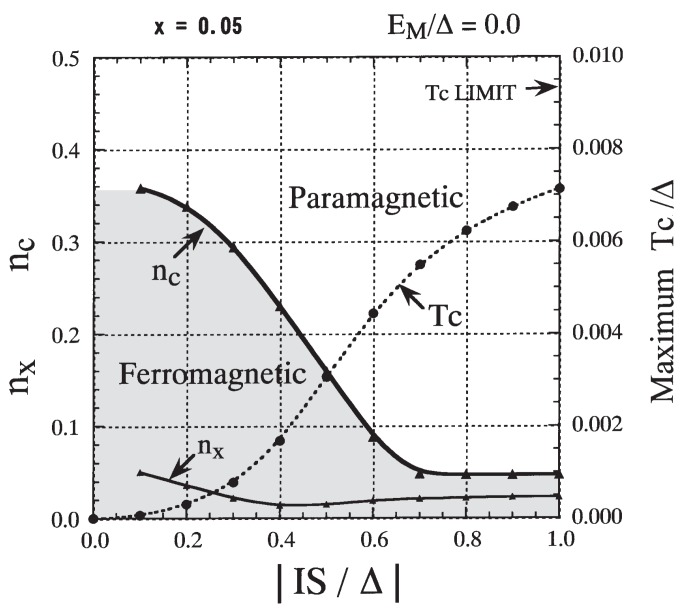
Phase diagram for $E_{M}=0$ and x=0.05. The critical value $n_{c}$ (solid line; left scale) and the maximum $T_{c}$ (dotted line; right scale) are presented as functions of |IS|/Δ. The carrier density $n_{x}$ at which $T_{c}$ reaches the maximum is included (solid line; left scale). From Takahashi and Kubo [42].

First we investigate the case of $E_{M}=0$. In Figure 9 the result for $T_{c}/\Delta$ is presented as a function of *n* for various values of IS/Δ. We immediately notice that there are two different types of behavior of $T_{c}$ as a function of *n* depending on the size of |IS|/Δ. When |IS|/Δ is small (|IS|/Δ≲0.3), ferromagnetism occurs over a wide range of *n*. The Curie temperature gradually increases with the increase in *n* and reaches a broad maximum. Then it gently decreases and vanishes at a critical value $n_{c}$. The maximum $T_{c}$ stays at a low value, and $n_{c}$ is much larger than x(=0.05). On the other hand, when |IS|/Δ is large (|IS|/Δ≳0.7), ferromagnetism occurs in a narrow range of n(≲x). $T_{c}$ rises steeply and reaches a maximum at $n_{x}\approx x/2$, and then it decreases rapidly. The maximum $T_{c}$ is high and $n_{c}$ is slightly less than *x*. These two different features can also be seen clearly in Figure 10, where $n_{c}$ and the maximum $T_{c}$ are depicted as functions of *n*. The carrier density $n_{x}$ at which $T_{c}$ reaches the maximum is also shown. Two different characteristic features were recognized in the phase diagrams obtained in earlier studies by Chattopadhyay *et al.* [51], Yagi and Kayanuma [52], and Calderón *et al.* [53].

When |IS|/Δ≲0.3, the maximum $T_{c}$ is approximately proportional to ${\left(IS/\Delta \right)}^{2}$. This suggests that in the range of |IS|/Δ≲0.3 the perturbative treatment of IS/Δ is available and that a Ruderman-Kittel-Kasuya-Yosida (RKKY)-like mechanism is expected to operate for a moderate carrier density. In order to clarify the properties of ferromagnetism that occurs when |IS|/Δ is small enough, we show the explicit expression for $T_{c}$ using the mean field approach. Assuming spin-split $\pm xI\langle S_{z}\rangle$ for the model band, we estimate the gain in total energy $E\left(0\right)-E(\langle S_{z}\rangle )$ to be $\rho \left(\varepsilon_{F}\right){\left(xI\langle S_{z}\rangle \right)}^{2}$, where $\rho (\varepsilon_{F})$ is the DOS at the Fermi level $\varepsilon_{F}$. Consequently, the $T_{c}$ is obtained as

(48) $$\begin{array}{ccc} k_{B}T_{c} & = & \frac{2}{3}x\rho \left(\varepsilon_{F}\right){\left(IS\right)}^{2}\; \end{array}$$

With a further increase in |IS|/Δ, the maximum $T_{c}$ rises rapidly (0.3≲|IS|/Δ≲0.7) and then tends to saturate. For |IS|/Δ≳0.7, ferromagnetism is induced only when n≲x and the maximum $T_{c}$ occurs at $n_{x}≅x/2$. The case with IS=−Δ shown in Figure 4 belongs to this region.

**Figure 11:**
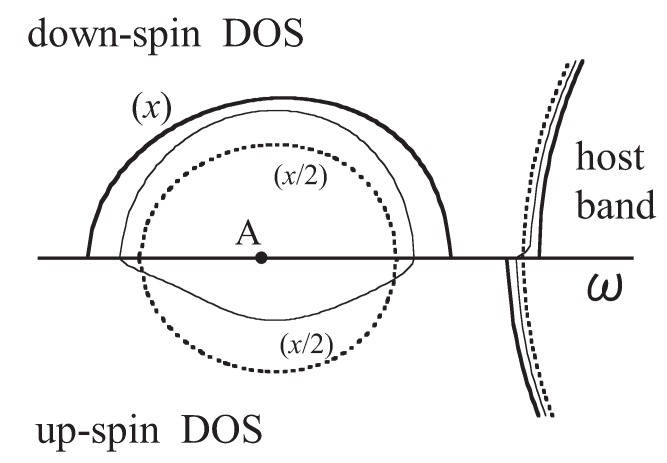
The DOS of the impurity band in the case of IS/Δ=−1.0 and $E_{M}=0$. The thick, thin, and dotted lines represent the cases of $\langle S_{z}\rangle /S=1.0$, 0.5, and 0.0, respectively. Dot A indicates the impurity level for x→0. From Takahashi and Kubo [41].

**Figure 12:**
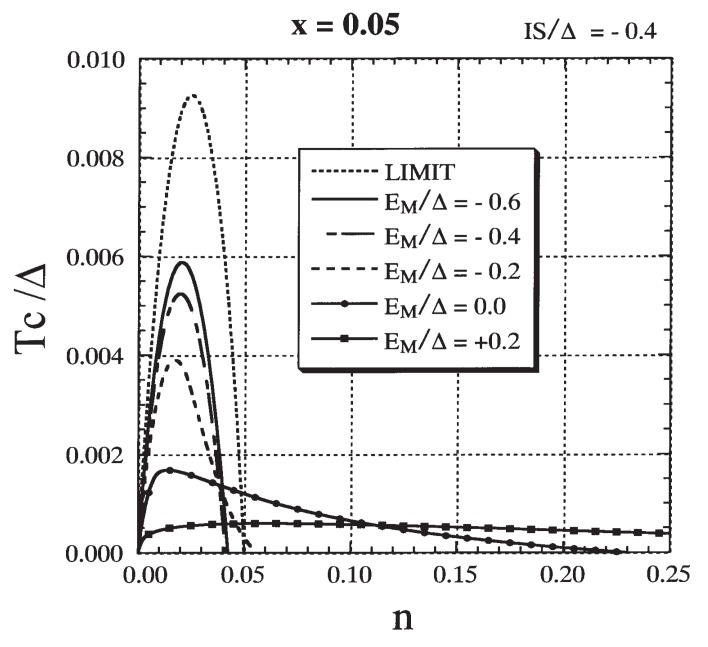
The result for $T_{c}/\Delta$ as a function of *n* for various values of $E_{M}/\Delta$ with x=0.05 and IS=−0.4Δ. The result denoted by `LIMIT’ is included (see text). From Takahashi and Kubo [42].

Here, we consider the mechanism of ferromagnetism that occurs in the magnetic impurity band of DMSs. In Figure 11, we extract the lower-energy part of the DOS from Figure 4. A magnetic impurity band forms around impurity level “A” and imitates the model band. The total number of states in the impurity band per site is equal to *x*, irrespective of the value of $\langle S_{z}\rangle$. When $\langle S_{z}\rangle =S$, all states in the impurity band are down-spin states, whereas when $\langle S_{z}\rangle =0$, the impurity band is composed of the same number of up- and down-spin states. The impurity band has a larger bandwidth in the ferromagnetic state than in the paramagnetic state. Hence, when the carrier concentration *n* is small, the ferromagnetic state has lower energy than the paramagnetic state. The energy gain initially increases with increasing *n* and reaches a maximum at n∼x/2. Then it gradually decreases and finally vanishes at n∼x. The gain of kinetic energy results in ferromagnetism below a certain temperature. This implies that a *double-exchange (DE)-like* mechanism for ferromagnetism is operative in the impurity band. Assuming that the impurity band has the same shape as the model DOS [defined by Equation (27)] in the limit of the strong exchange interaction, the bandwidth of the magnetic impurity band is estimated to be $2\sqrt{x}\Delta$ when $\langle S_{z}\rangle =S$ and $\sqrt{2x}\Delta$ when $\langle S_{z}\rangle =0$. The results for $T_{c}$ based on this assumption are inserted in Figure 9 and Figure 12 as “LIMIT”. The maximum $T_{c}$ is estimated to be

(49) $$\begin{array}{ccc} k_{B}T_{c} & = & \frac{2(2-\sqrt{2})}{9\pi }\sqrt{x}\Delta \; \end{array}$$

at n=x/2. The maximum $T_{c}\;\left(=0.0093\Delta \right)$ for x=0.05 is indicated by an arrow in Figure 10.

It is worth noting that the Zener double-exchange mechanism for ferromagnetism is usually understood to be effective only when the exchange energy is larger than the width of the carrier band (or |IS|≳2Δ) in the case where magnetic ions sit on every site [54]. In the present case, the exchange energy is not greater than the width of the model band. Nevertheless, a *DE-like* mechanism functions because the magnetic impurity bandwidth is smaller than the exchange energy (or $\left|IS\right|≳\sqrt{2x}\Delta$).

From the *n* dependence of $T_{c}$ shown in Figure 9, we conclude that the DE-like mechanism in a magnetic impurity band becomes dominant when |IS|/Δ≳0.7 if $E_{M}=0$. On the other hand, the DE-like mechanism is not relevant to the ferromagnetism in II-VI DMSs because the magnetic impurity level does not appear as illustrated in Figure 5 and Figure 6, in which the parameters |IS|/Δ=0.4 and $E_{M}=0$ were employed.

Next, we study the role of the attractive potential in order to elucidate the origin of the carrier-induced ferromagnetism in III-V-based DMSs. We have already pointed out the similarity of the lower-energy part of the DOSs between Figure 4 (strong exchange interaction) and Figure 8 (strong attractive potential). The similarity is due to the fact that the impurity level has the same energy, which is determined by the effective attractive potential $E_{M}+IS$. From the strong similarity in the low-energy part of the DOS, we may expect that ferromagnetism occurs through the same mechanism in both cases. In Figure 12 the effect of the nonmagnetic potential $E_{M}$ on $T_{c}$ is presented for IS fixed at −0.4Δ. The impurity level appears for $E_{M}<-0.1\Delta$ in this case. When $E_{M}≳0.0$ the $T_{c}$ stays low and $n_{c}$ is much larger than *x*, while for $E_{M}≲-0.2\Delta$ a high $T_{c}$ is realized and $n_{c}$ is less than *x*. In the latter region the DE-like mechanism becomes operative. The criterion for the DE-like mechanism to operate is roughly estimated to be $IS+0.4E_{M}≲-0.6\Delta$. The result suggests that the *DE-like* mechanism can be operative when an attractive potential assists in the production of an impurity band even though the exchange interaction is not particularly strong.

### 3.7. Specific Results for (Ga,Mn)As

Here we discuss (Ga,Mn)As, which has attracted much attention in recent years owing to its so-called carrier-induced ferromagnetism. Though the microscopic mechanism for carrier-induced ferromagnetism is still controversial, the following properties seem to be generally accepted for (Ga,Mn)As: (i) Mn ions substitute randomly for Ga cations in the zincblende structure [26]. (ii) A Mn ion in GaAs gives rise to an acceptor level at about 0.113 eV above the valence band [55]. (iii) The Mn ion has highly localized *d* states with a magnetic moment of $∼5\mu_{B}$ (or S=5/2) [55,56,57]. (iv) The Mn-induced states near the Fermi energy play a key role in the origin of ferromagnetism. According to photoemission studies [58,59,60], X-ray absorption spectroscopy [61], and band calculations [62,63], these states are mainly created in As 4*p* orbits. (v) The *p-d* exchange interaction between the As 4*p* hole and the localized *d* spin is antiferromagnetic [64,65], and its amplitude is not very different from that in II-VI-based DMSs [60,66]. (vi) As antisite defects (As ions sitting on Ga lattice site) and Mn interstitials are common in semiconductor samples grown by low-temperature molecular beam epitaxy [27,67,68,69,70].

Mn interstitials act as double donors. Many holes may be trapped not at Mn acceptors but at such defects, although we may expect that one hole is donated by a Mn atom. The density of the holes and that of the Mn ions are therefore regarded as separate sample-dependent quantities that are to be determined experimentally.

**Figure 13:**
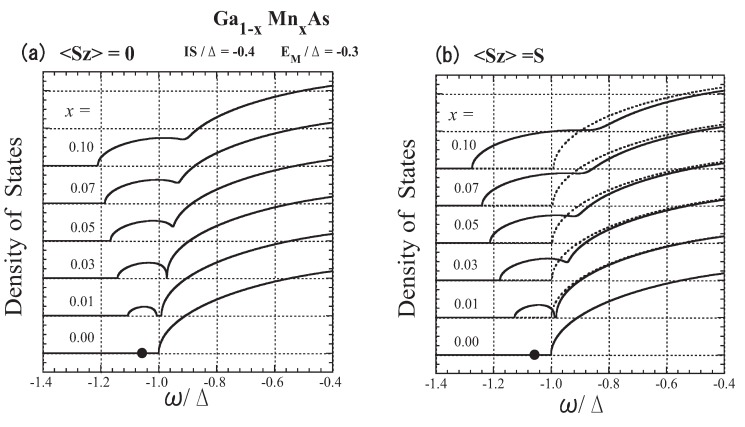
The lower-energy part of the DOS of Ga${}_{1-x}$Mn${}_{x}$As for various values of *x*: (a) $\langle S_{z}\rangle =0$ and (b) $\langle S_{z}\rangle =0$. The solid lines represent the down-spin carrier and the dotted lines represent the up-spin carrier. The impurity level $E_{a}=-1.057\Delta$ is indicated by a dot on the line x=0. From Takahashi and Kubo [42].

For Ga${}_{1-x}$Mn${}_{x}$As, we set the (*p*-) bandwidth 2Δ=4 eV from the band calculations [62,63], and take IS=−0.4Δ, which corresponds to $N_{0}\beta =-0.64$ eV. Then we determine $E_{M}$ to be −0.3Δ so as to yield the acceptor energy of 0.113 eV (=0.057Δ)[55]. The *x* dependence of the DOS is shown in Figure 13; $\langle S_{z}\rangle =0$ (a) and $\langle S_{z}\rangle =S$ (b). The present model parameters lead to an impurity level at the energy of $E_{a}=-1.057\Delta$ in the dilute limit (x→ 0). With an increase in *x*, an impurity band forms, and for x≳0.02 it merges into the host valence band. The results consistently explain the experimental observation of impurity-band-like states [59,71] and the insulator-metal transition at x∼0.03 [72,73].

**Figure 14:**
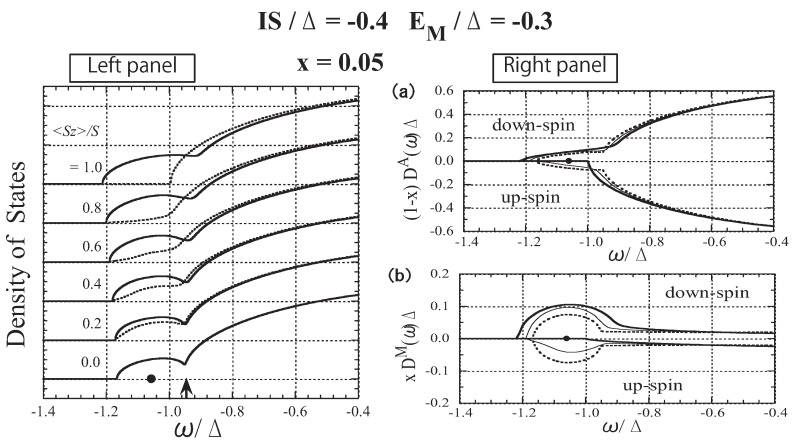
The results for Ga${}_{1-x}$Mn${}_{x}$As with x=0.05. Left panel: Low-energy part of the DOS shown for various values of $\langle S_{z}\rangle /S$. The solid lines represent the down-spin carrier and the dotted lines represent the up-spin carrier. The arrow indicates the Fermi level for n=x(=0.05). The impurity level $E_{a}=-1.057\Delta$ is indicated by a dot on the horizontal line $\langle S_{z}\rangle =0$. Right panel: (a) Ga-site component of the DOS, $\left(1-x\right)D_{↓}^{A}\left(\omega \right)\Delta$ and $-\left(1-x\right)D_{↑}^{A}\left(\omega \right)\Delta$ and (b) Mn-site component of the DOS, $xD_{↓}^{M}\left(\omega \right)\Delta$ and $-xD_{↑}^{M}\left(\omega \right)\Delta$. The thick, thin, and dotted lines represent the cases of $\langle S_{z}\rangle /S=1.0,0.5$, and 0.0, respectively. From Takahashi and Kubo [42].

**Figure 15:**
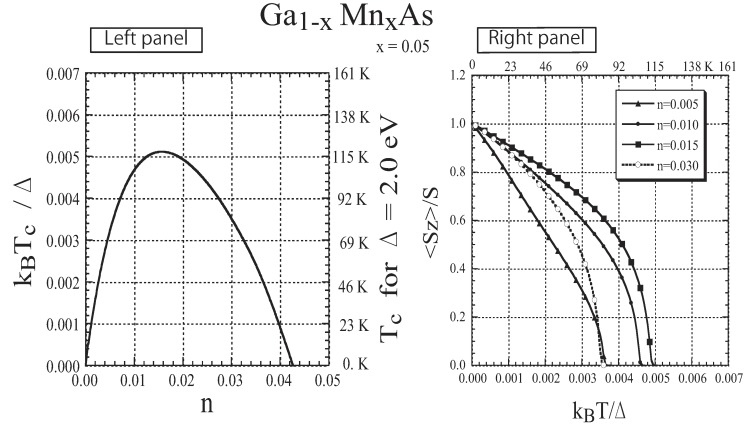
The results for Ga${}_{1-x}$Mn${}_{x}$As with x=0.05. Left panel: Curie temperature $T_{c}$ as a function of *n*. Right panel: Magnetization as a function of the temperature *T* for various values of *n*. From Takahashi and Kubo [42] and Takahashi *et al.* [44].

In Figure 14 and Figure 15, the results for x=0.05 are presented. The lower band tail shown in the left panel of Figure 14 is strongly affected by the change in $\langle S_{z}\rangle$. The results of species-resolved analysis shown in Figure 14a,b reveal that the change in the band tail is mainly ascribed to the change in the local DOS at the Mn site. This result indicates that a carrier at the band tail usually stays at Mn sites in spite of the small x(=0.05). The present result is in sharp contrast with the free-carrier picture, which is the premise for the application of the RKKY model [74], but is consistent with the nearly bound hole picture deduced on the basis of infrared optical absorption measurement [75,76]. The carrier’s spin is tightly coupled with the localized spin. In the left panel of Figure 15, we plot the Curie temperature $T_{c}$ as a function of *n*. It has been reported that ferromagnetism with $T_{c}=110$ K is realized in Ga${}_{1-x}$Mn${}_{x}$As with x=0.053 when *n* is 30% of the nominal concentration (*x*) of Mn [26]. The agreement of the present result with the experimental observation is satisfactory. The *T* dependence of the magnetization is presented in the right panel of Figure 15 for various values of *n*. The present result is consistent with the experimentally obtained magnetization [26]. We have also verified that the result obtained by applying DMFT to the present model is almost the same as the present result [44].

Here, we briefly discuss the origin and the mechanism of the carrier-induced ferromagnetism of (Ga,Mn)As. The results for the *n* dependence of $T_{c}$ shown in the left panel of Figure 15 are well explained by the *DE-like* mechanism described above. Although the impurity band is not separated in the case of x≃0.05, the carriers in the band tail of Ga${}_{1-x}$Mn${}_{x}$As have such a high local carrier density at Mn sites that the carrier spins couple strongly to the fluctuating localized spins. Thus, the hopping of carriers among Mn sites causes the ferromagnetic ordering of the localized spins through the *DE-like* mechanism.

Since Zener originally proposed the DE interaction for (La,Ca)MnO${}_{3}$, where 3*d* holes hop among the magnetic ions located at the regular lattice sites [54], it might be understood that the DE mechanism is only relevant to the hopping of 3*d* holes in (Ga,Mn)As [77]. The DE mechanism for ferromagnetism, in fact, works quite generally. The only condition required for the mechanism is very strong spin coupling between carrier spins and localized spins. If this is satisfied, carriers may have any character and the localized spins can be arranged randomly. In the case of III-V-based DMSs, carriers are considered to have 4*p* character [60,61] and the strength of the *p-d* exchange interaction is not very different from that in II-VI-based DMSs [66]. The Coulomb interaction between the carrier (hole) and a Mn${}^{2+}$ ion (acceptor center), however, promotes the formation of a magnetic impurity band, and strong spin coupling is realized in the magnetic impurity band and/or in the band tail. Therefore, the DE mechanism induces ferromagnetism. We call this mechanism the *DE-like* mechanism to avoid confusion with the argument assuming *d* holes [77]. Note that no Mn${}^{3+}$ ($d^{4}$ configuration) states have been experimentally detected in (Ga,Mn)As [55,57,60,64,78]. All these experimental observations suggest that the fixed valence state Mn${}^{2+}$ (S=5/2) is realized in (Ga,Mn)As.

**Figure 16:**
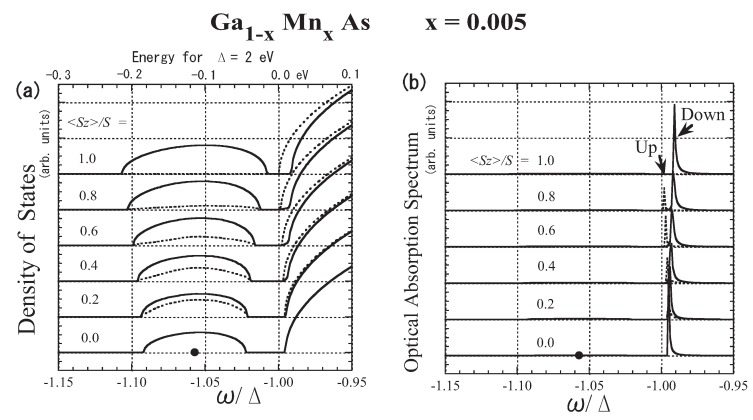
The result for Ga${}_{1-x}$Mn${}_{x}$As with x=0.005: (a) low-energy part of the DOS D(ω), (b) optical absorption band A(ω) in arbitrary units (arb. units). The solid lines represent the down-spin carrier and the dotted lines represent the up-spin carrier. Note that the energy of the bottom of the model band is ω=−Δ. Along the upper horizontal axis of (a), energies for Δ=2 eV are graduated in eV, where the origin of the energy, 0.0 eV, is taken at ω=−Δ. The impurity level $E_{a}=-1.057\Delta$ (or −0.113 eV) is indicated by a dot on the line $\langle S_{z}\rangle =0$. From Takahashi [43].

Although the exchange interaction between *p* holes and *d* spins has been experimentally proved to be antiferromagnetic [60,64,65], in the early stage of research, the ferromagnetic exchange interaction was reported on the basis of polarized magnetoreflection measurement [79]. When an impurity band exists, the optically observed band edge is not necessarily the band edge of the impurity band. The present result for x=0.005 is shown in Figure 16. When x=0.5%, the impurity band is separate from the host band, as shown in Figure 16(a). The optical absorption spectrum corresponds to the k=0 component of the DOS. Therefore, the optical absorption spectrum takes negligible values in the impurity band, because the impurity band is constituted from the wide range of *k* space. The optical band edge, $\omega_{p}$, lies almost at the bottom of the *host* band. Thus, although a negative IS is assumed, the optical band edge with up (down) spin shifts to the low (high)-energy side with increasing $\langle S_{z}\rangle$, as shown in Figure 16(b). Hence, the direction of the shift in the optical band edge is opposite to the direction obtained by the VCA. Note that ferromagnetic spin coupling is realized near the bottom of the host band edge. In magnetoreflection measurement, not the shift of the impurity band edge but the shift of the host band edge was detected. A simple calculation based on the present approach with $N_{0}\beta =-0.64$ eV predicts that the magnetoreflection measurement will deduce the apparent value of $N_{0}\beta$ to be +1.3 eV, which is consistent with the experimental observation [79].

**Figure 17:**
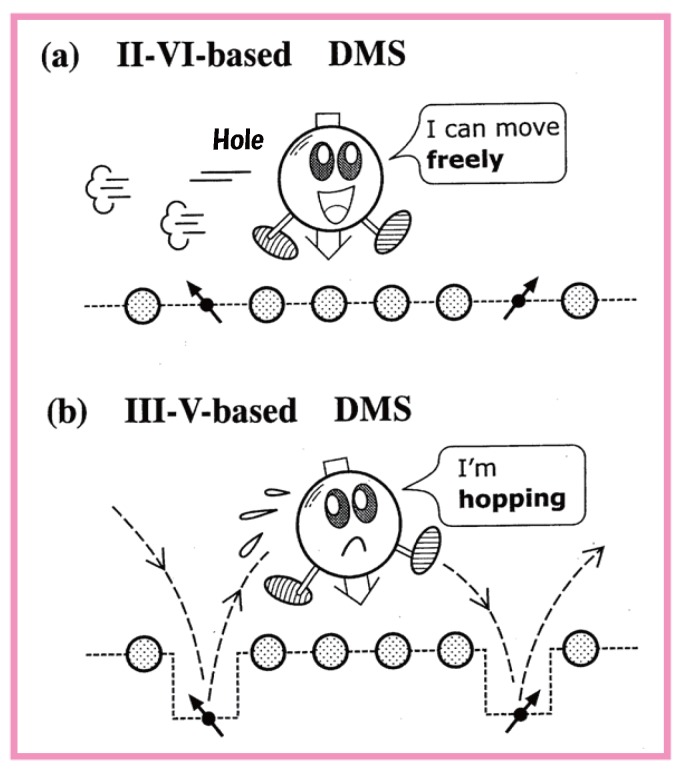
An illustration of the difference between the characters of a hole in a II-VI-based DMS and in a III-VI-based DMS.

The difference between the characters of a hole in a II-VI-based DMS and a hole in a III-VI-based DMS is illustrated in Figure 17. The hole in a II-VI-based DMS can move freely over many sites while undergoing exchange interactions with the *d* spin on Mn sites. On the contrary, the hole in a III-V-based DMS moves while hopping from a Mn site to another Mn site because the attractive Coulomb potential makes it favorable for the hole to remain at Mn sites.

## 4. Summary and Concluding Comments

Throughout this review article, we considered a simple model with fewer physical parameters [80]. The advantage of this approach is that the basic physics of systems can be explained in a simple way, regardless of the strong material dependence, although realistic electronic-structure calculations such as first-principles calculations based on density functional theory [16,17,81,82] or numerical methods such as the quantum Monte Carlo method [83,84] may give more realistic results.

First, applying the dynamical CPA to the *s*-*f* model, we showed the numerical results for FMSs such as EuO and EuS. The results for the DOS and the energy of the bottom of the band were given for various exchange energies and temperatures. Not only the dependence of the band edge on the temperature but also the magnitude of the redshift can be explained satisfactorily. We pointed out that the electron-spin polarization cannot reach 100% due to the quantum effect coming from the finiteness of the magnitude of the *f* spin [34]. We also add that the temperature dependence of the electrical resistivity of a degenerate FMS can be consistently explained except around $T_{c}$ [35]; the dynamical CPA becomes inefficient when the short-range order of *f* spins develops near $T_{c}$ [85,86,87,88] .

Next, we considered a simple model for carriers in the A${}_{1-x}$Mn${}_{x}$B-type DMS. The model includes not only the exchange interaction but also the nonmagnetic local potential at the magnetic Mn ion site. Based on the results obtained by applying the dynamical CPA to the simple model, we discussed the carrier states of three typical cases: cases with strong and moderate exchange interactions in the absence of nonmagnetic potentials, and the case with strong attractive nonmagnetic potentials in addition to moderate exchange interaction. We showed that the mechanism of carrier-induced ferromagnetism changes from the RKKY-type mechanism to the *DE-like* mechanism with the increase in the amplitude of the exchange interaction. Furthermore, the *DE-like* mechanism can be operative when an attractive potential assists in the production of an impurity band even though the exchange interaction is not particularly strong.

Carrier states in II-VI-based DMSs are well described by the present model with moderate exchange interaction. The results obtained by applying the dynamical CPA to the model explain the anomalous *x* dependence (*i.e.*, bowing) of the energy gap of wide-band-gap II-VI-based DMSs [38], the apparent enhancement of the *p*-*d* exchange interaction, and the asymmetric splitting of exciton states in Cd${}_{1-x}$Mn${}_{x}$S [39,40]; these effects cannot be explained by the VCA.

In the case of III-VI-based DMSs such as Ga${}_{1-x}$Mn${}_{x}$As and In${}_{1-x}$Mn${}_{x}$As, Coulomb attractive potentials assist the formation of the impurity band and/or band tail and promote the *DE-like* mechanism although the exchange interaction is not sufficiently strong. Setting the parameters so as to yield an experimentally observed impurity level, we calculated the DOS for various *x* and magnetizations, as well as the dependence of $T_{c}$ on *n*. The results for the local DOS suggest that the exchange coupling between a carrier and localized spins at Mn sites is very strong [41,42]. Thus, we conclude that the carrier-induced ferromagnetism of Ga${}_{1-x}$Mn${}_{x}$As is ascribed to a *DE-like* mechanism realized in the magnetic impurity band/or in the band tail. The present results also reveal the difference between the optical band edges in II-VI-based DMSs and in III-V-based DMSs [43].

However, we have to point out that the many-body effect [89] and clustering effect [90] are not considered in our CPA. With the increase in Mn fraction *x*, the effects of the direct antiferromagnetic (AF) superexchange interaction between neighboring Mn impurities become important [90]. It should also be considered that the effect of the Coulomb attractive potential $E_{M}$ becomes less important as the carrier density increases [45]. As noted in Section 3.7, in real samples of (Ga,Mn)As, Mn ions do not always substitute for Ga cations. Some fraction of Mn may reside in the interstitial lattice sites and act as double donors [68]; many holes may be trapped at such defects, reducing the concentration of holes. Furthermore, As antisites may induce a so-called disordered local moment configuration, where only part of the randomly distributed Mn atoms are ferromagnetically aligned while the rest of the Mn atoms have magnetic moments oriented antiparallel to each other [69]. These effects are beyond the scope of our simple model and approach.

Recently, Gd-doped EuO has been highlighted for potential use in spintronics devices owing to its with high $T_{c}$ [5,6,8]. The mechanism of the enhancement of $T_{c}$ of Gd-doped EuO is a controversial problem. This problem can be treated by applying the dynamical CPA to a simple model adapted for Eu${}_{1-x}$Gd${}_{x}$O. The metal-insulator transition in Eu-rich EuO is still under discussion [15]. The model for Eu${}_{1-y}$V${}_{y}$O, where V denotes a vacancy of an O ion, is the same as that for A${}_{1-x}$Mn${}_{x}$B-type DMSs, and can act as a substitute for EuV${}_{y}$O${}_{1-y}$, which is the most realistic model of Eu-rich EuO. An extension of the dynamical CPA to these problems is now in progress and will be published separately.

## Appendix

### Dynamical CPA–Locator Formalism

In the Appendix, we briefly outline an alternative but equivalent condition of the CPA, which is an extension of the CPA using the locator formalism [32]. Assuming that the spin-dependent effective medium surrounds an arbitrary site *n*, we consider the transfer of the carrier with spin *μ* between site *n* and the effective medium (*i.e.*, $\Sigma_{↑}$ and $\Sigma_{↓}$) by the site-renormalized interactor $J_{\mu }$. Then, the propagator $G^{A}\;\left(G^{M}\right)$ associated with the real potential of $u_{n}^{A}\;\left(u_{n}^{M}\right)$ embedded at site *n* in the medium is defined by

(50a) $$\begin{array}{ccc} G^{A} & = & \frac{1}{\omega -u_{n}^{A}-\sum_{\mu }J_{\mu }a_{n\mu }^{†}a_{n\mu }}\; \end{array}$$

(50b) $$\begin{array}{ccc} G^{M} & = & \frac{1}{\omega -u_{n}^{M}-\sum_{\mu }J_{\mu }a_{n\mu }^{†}a_{n\mu }}\; \end{array}$$

When we set the coherent potential $\Sigma_{\mu }$ at site *n* in the effective medium, the reference propagator,

(51) $$\begin{array}{ccc} P & = & \frac{1}{\omega -\sum_{\mu }\Sigma_{\mu }a_{n\mu }^{†}a_{n\mu }-\sum_{\mu }J_{\mu }a_{n\mu }^{†}a_{n\mu }}\; \end{array}$$

is equivalent to the Green function for the effective medium. Thus, the diagonal matrix element of *P* is equal to $F_{\mu }$ defined by Equation (15):

(52) $$\begin{array}{ccc} F_{\mu }\left(\omega \right) & = & \langle n\mu |P|n\mu \rangle =\frac{1}{\omega -\Sigma_{\mu }-J_{\mu }}\; \end{array}$$

Equation (52) gives the relationship between $J_{\mu }$ and $F_{\mu }$; $L_{\mu }\equiv 1/\left(\omega -\Sigma_{\mu }\right)$ is called a locator. Hereafter, for the sake of simplicity, the site-diagonal elements in the Wannier representation $\langle n\mu |G^{A}|n\nu \rangle$ are written as $G_{\mu \nu }^{A}$ (μ,ν=↑, or ↓).

Then, the spin-diagonal element of $G^{A}$ is given by

(53) $$\begin{array}{ccc} F_{\mu }^{A}\left(\omega \right)=G_{\mu \mu }^{A} & = & \frac{1}{\omega -E_{A}-J_{\mu }}\; \end{array}$$

and the spin-off-diagonal elements are $G_{↑↓}^{A}=G_{↓↑}^{A}=0$.

In order to obtain the explicit expression for the site-diagonal elements of $G^{M}$, we rewrite Equation (50b) as

(54) $$\begin{array}{ccc} G^{M}\left(\omega -u_{n}^{M}-\sum_{\mu }J_{\mu }a_{n\mu }^{†}a_{n\mu }\right) & = & 1\; \end{array}$$

Equation (54) is written in the spin-matrix-element expression as

(55a) $$\begin{array}{ccc} G_{↑↑}^{M}\left(\omega -E_{M}+IS_{z}-J_{↑}\right)+G_{↑↓}^{M}\left(IS_{+}\right) & = & 1\; \end{array}$$

(55b) $$\begin{array}{ccc} G_{↑↑}^{M}\left(IS_{-}\right)+G_{↑↓}^{M}\left(\omega -E_{M}-IS_{z}-J_{↓}\right) & = & 0\; \end{array}$$

Equation (55) is the simultaneous equations concerning $G_{↑↑}^{M}$ and $G_{↑↓}^{M}$, which can be solved after a somewhat complicated calculation using the commutation relationships between the components of S (but with no further approximations). The resulting expressions are

(56a) $$\begin{array}{ccc} G_{↑↑}^{M} & = & \frac{\omega -E_{M}-I\left(S_{z}+1\right)-J_{↓}}{\left[\omega -\left(E_{M}-IS_{z}\right)-J_{↑}\right]\left[\omega -E_{M}-I\left(S_{z}+1\right)-J_{↓}\right]-I^{2}\left[S\left(S+1\right)-S_{z}^{2}-S_{z}\right]} \end{array}$$

(56b) $$\begin{array}{ccc} G_{↓↓}^{M} & = & \frac{\omega -E_{M}+I\left(S_{z}-1\right)-J_{↑}}{\left[\omega -\left(E_{M}+IS_{z}\right)-J_{↓}\right]\left[\omega -E_{M}+I\left(S_{z}-1\right)-J_{↑}\right]-I^{2}\left[S\left(S+1\right)-S_{z}^{2}+S_{z}\right]} \end{array}$$

(56c) $$\begin{array}{ccc} G_{↑↓}^{M} & = & \frac{1}{\left[\omega -\left(E_{M}-IS_{z}\right)-J_{↑}\right]\left[\omega -E_{M}-I\left(S_{z}+1\right)-J_{↓}\right]-I^{2}\left[S\left(S+1\right)-S_{z}^{2}-S_{z}\right]}\left(-IS_{-}\right) \end{array}$$

(56d) $$\begin{array}{ccc} G_{↓↑}^{M} & = & \frac{1}{\left[\omega -\left(E_{M}+IS_{z}\right)-J_{↓}\right]\left[\omega -E_{M}+I\left(S_{z}-1\right)-J_{↑}\right]-I^{2}\left[S\left(S+1\right)-S_{z}^{2}+S_{z}\right]}\left(-IS_{+}\right)\; \end{array}$$

Note that the site-diagonal elements of $G^{M}$ involve spin operators. Thus, $F_{\mu }^{M}\left(\omega \right)$ is defined as the thermal average of the spin-diagonal element $G_{\mu \mu }^{M}$ by

(57) $$F_{\mu }^{M}\left(\omega \right)=\langle G_{\mu \mu }^{M}\rangle =\sum_{S_{z}=-S}^{S}G_{\mu \mu }^{M}\left(S_{z}\right)\exp\left(\lambda S_{z}\right)/\sum_{S_{z}=-S}^{S}\exp\left(\lambda S_{z}\right)\;$$

where $\lambda \;(\equiv h/k_{B}T)$ is determined so as to reproduce a given value of $\langle S_{z}\rangle$ [see Equation (24)]. Note that the spin-off-diagonal elements $\langle G_{↑↓}^{M}\rangle =\langle G_{↓↑}^{M}\rangle =0$, because $G_{↑↓}^{M}$ ($G_{↓↑}^{M}$) includes $S_{-}$ ($S_{+}$) in its final form. Finally, the CPA condition in the locator formula is given by

(58) $$\begin{array}{ccc} F_{\mu }\left(\omega \right) & = & \left(1-x\right)F_{\mu }^{A}\left(\omega \right)+xF_{\mu }^{M}\left(\omega \right) \end{array}$$

When $F_{\mu }$ is given, $J_{\mu }$ is calculated by Equations (29) and (52) [*i.e.*, $J_{\mu }=\left(\Delta^{2}/4\right)F_{\mu }$ for the model band defined by Equation (27)]. Then, $F_{\mu }^{A}$ and $F_{\mu }^{M}$ are calculated by Equations (53) and (57), respectively, and consequently, $F_{\mu }$ is again obtained by Equation (58). Therefore, $F_{\mu }$ and $J_{\mu }$ are determined self-consistently.

The advantage of the locator formula of the CPA is that it is straightforward to determine the species-resolved DOS, *i.e.*, the DOS associated with each type of ion in the alloy. The local DOS at the A(M) site is obtained by

(59a) DμA(ω)=−1πImFμA(ω)

(59b) DμM(ω)=−1πImFμM(ω)

In actual calculations, we first determined $F_{\mu }$ and $\Sigma_{\mu }$ by the *t*-matrix formula of the CPA, then calculated $J_{\mu }$, and finally calculated $D_{\mu }^{A}\left(\omega \right)$ and $D_{\mu }^{M}\left(\omega \right)$. We verified the relations

(60) $$\begin{array}{c} \int_{-\infty }^{\infty }D_{\mu }^{A}\left(\omega \right)d\omega =\int_{-\infty }^{\infty }D_{\mu }^{M}\left(\omega \right)d\omega =1\; \end{array}$$

and

(61) $$\begin{array}{c} D_{\mu }\left(\omega \right)=\left(1-x\right)D_{\mu }^{A}\left(\omega \right)+xD_{\mu }^{M}\left(\omega \right)\; \end{array}$$

which are a consequence of the CPA condition in the locator formula, Equation (58).

## References

[1] Wacter P., Gschneidner K.A., Eyring L. (1979). Europium chalcogenides: EuO, EuS, EuSe and EuTe. Handbook on Physics and Chemistry of Rare Earths.

[2] Mauger A., Godart C. (1986). The magnetic, optical, and transport properties of representatives of a class of magnetic semiconductors: The europium chalcogenides. Phys. Rep..

[3] von Molnar S., Kasuya T. (1968). Evidence of band conduction and critical scattering in dilute Eu-chalcogenide alloys. Phys. Rev. Lett..

[4] Oliver M.R., Dimmock J.O., McWhorter A.L., Reed T.B. (1971). Conductivity studies in europium oxide. Phys. Rev. B.

[5] Matsumoto T., Yamaguchi K., Yuri M., Kawaguchi K., Koshizaki N., Yamada K. (2004). Preparation of Gd-doped EuO_1−__*x*_ thin films and the magnetic and magneto-transport properties. J. Phys-Condens. Matter.

[6] Ott H., Heise S.J., Sutarto R., Hu Z., Chang C.F., Hsieh H.H., Lin H.-J., Chen C.T., Tjeng L.H. (2006). Soft x-ray magnetic circular dichroism study on Gd-doped EuO thin films. Phys. Rev. B.

[7] Santos T.S., Moodera J.S., Raman K.V., Negusse E., Holroyd J., Dvorak J., Liberati M., Idzerda Y.U., Arenholz E. (2008). Determining exchange splitting in a magnetic semiconductor by spin-filter tunneling. Phys. Rev. Lett..

[8] Sutarto R., Altendorf S.G., Coloru B., Moretti Sala M., Haupricht T., Chang C.F., Hu Z., Schusler-Langeheine C., Hollmann N., Kierspel H., Mydosh J.A., Hsieh H.H., Lin H.-J., Chen C.T., Tjeng L.H. (2009). Epitaxy, stoichiometry, and magnetic properties of Gd-doped EuO films on YSZ (001). Phys. Rev. B.

[9] Kimura S., Ito T., Miyazaki H., Mizuno T., Iizuka T., Takahashi T. (2008). Electronic inhomogeneity EuO: Possibility of magnetic polaron states. Phys. Rev. B.

[10] Comment A., Ansermet J.-P., Slichter C.P., Rho H., Snow C.S., Cooper S.L. (2005). Magnetic properties of pure and Gd-doped EuO probed by NMR. Phys. Rev. B.

[11] Miyazaki H., Ito T., Im H.J., Yagi S., Kato M., Soda K., Kimura S. (2009). Direct observation of momentum-dependent exchange interaction in a Heisenberg ferromagnet. Phys. Rev. Lett..

[12] Steeneken P.G., Tjeng L.H., Elfimov I., Sawatzky G.A., Ghiringhelli G., Brookes N.B., Huang D.-J. (2002). Exchange splitting and charge carrier spin polarization in EuO. Phys. Rev. Lett..

[13] Souza-Neto N.M., Haskel D., Tseng Y.-C., Lapertot G. (2009). Pressure-induced electronic mixing and enhancement of ferromagnetic ordering in EuX (X=Te, Se, S, O) magnetic semiconductors. Phys. Rev. Lett..

[14] Schiller R., Müller W., Nolting W. (2001). Kondo-lattice model: Application to the temperature-dependent electronic structure of EuO(100) films. Phys. Rev. B.

[15] Sinjukow P., Nolting W. (2004). Fully self-consistent determination of transport properties in Eu-rich EuO. Phys. Rev. B.

[16] Ghosh D.B., De M., De S.K. (2004). Electronic structure and magneto-optical properties of magnetic semiconductors: Europium monochalcogenides. Phys. Rev. B.

[17] Ingle N.J.C., Elfimov I.S. (2008). Influence of epitaxial strain on the ferromagnetic semiconductor EuO: First-principles calculations. Phys. Rev. B.

[18] Arnold M., Kroha J. (2008). Simultaneous ferromagnetic metal-semiconductor transition in electron-doped EuO. Phys. Rev. Lett..

[19] Nolting W., Java M.S., Rex S. (1996). Magnetic polaron in ferro- and antiferromagnetic semiconductors. Phys. Rev. B.

[20] Nolting W., Rex S., Java M.S. (1997). Magnetism and electronic structure of local moment ferromagnet. J. Phys-Condens. Matter.

[21] Bryksa V., Nolting W. (2008). Disordered Kondo-lattice model: Extension of coherent potential approximation. Phys. Rev. B.

[22] Tang G., Nolting W. (2007). Carrier-induced ferromagnetism in diluted local moment systems. Phys. Rev. B.

[23] Furdya J.K. (1988). Diluted magnetic semiconductors. J. Appl. Phys..

[24] Haas K.C., Averous M., Balkanski M. (1991). Band structure and theory of magnetic interactions. Semimagnetic Semiconductors and Diluted Magnetic Semiconductors.

[25] Lascaray J.P., Averous M., Balkanski M. (1991). II_1−*x*_Mn_*x*_VI semimagnetic semiconductors. Semimagnetic Semiconductors and Diluted Magnetic Semiconductors.

[26] Ohno H. (1999). Properties of ferromagnetic III-V semiconductors. J. Magn. Magn. Mater..

[27] Jungwirth T., Masek J., Kucera J., MacDonald H. (2006). Theory of ferromagnetic (III,Mn)V semiconductors. Rev. Mod. Phys..

[28] Ehrenreich H., Schwartz L.M., Ehrenreich H., Seitz F., Turnbull D. (1976). The electronic structure of alloys. Solid State Physics, Advances in Research and Application.

[29] Gonis A., van Groesen E., de Jager E.M. (1992). Green functions for ordered and disordered systems. Studies in Mathematical Physics.

[30] Onodera Y., Toyozawa Y. (1968). Persistence and amalgamation types in the electronic structure of mixed crystals. J. Phys. Soc. Jpn..

[31] Rangette A., Yanase A., Kübker J. (1973). CPA treatment of the *s*-*d* model at high temperatures. Solid State Commun..

[32] Kubo K. (1974). Electronic states in magnetic semiconductors–An extension of CPA to random spin systems–. J. Phys. Soc. Jpn..

[33] Takahashi M., Mitsui K. (1996). Single-site approximation for the *s*-*f* model in ferromagnetic semiconductors. Phys. Rev. B.

[34] Takahashi M. (1997). Electron-spin polarization in ferromagnetic semiconductors. Phys. Rev. B.

[35] Takahashi M., Mitsui K. (1998). Electron scattering due to fluctuating localized spins in degenerate ferromagnetic semiconductors. J. Magn. Magn. Mater..

[36] Takahashi M. (1997). Conduction electron band in antiferromagnetic semiconductors. Phys. Rev. B.

[37] Takahashi M., Nolting W. (2001). Single-site approximation for the *s*-*f* model of antiferromagnetic semiconductors. J. Magn. Magn. Mater..

[38] Takahashi M. (1999). Coherent-potential approach to magnetic and chemical disorder in diluted magnetic semiconductors. Phys. Rev. B.

[39] Takahashi M. (2001). Coherent potential approach to exchange-induced band splitting in diluted magnetic semiconductors under a saturating magnetic field. J. Phys-Condens. Matter.

[40] Takahashi M. (2001). Asymmetric splitting of exciton states in diluted magnetic semiconductor Cd_1−*x*_Mn_*x*_S. J. Phys. Soc. Jpn..

[41] Takahashi M., Kubo K. (2002). Mechanism of carrier-induced ferromagnetism in magnetic semiconductors. Phys. Rev. B.

[42] Takahashi M., Kubo K. (2003). Carrier states and ferromagnetism in diluted magnetic semiconductors. J. Phys. Soc. Jpn..

[43] Takahashi M. (2004). Optical band edge of diluted magnetic semiconductors. Phys. Rev. B.

[44] Takahashi M., Furukawa N., Kubo K. (2003). Mechanism of carrier-induced ferromagnetism in diluted magnetic semiconductors. J. Magn. Magn. Mater..

[45] Popescu F., Şen C., Dagotto E., Moreo A. (2007). Crossover from impurity to valence band in diluted magnetic semiconductors: Role of Coulomb attraction by acceptors. Phys. Rev. B.

[46] Hoang A.T. (2008). Optical properties of diluted magnetic semiconductors in coherent potential approximation. Physica B.

[47] Nolting W., Oreś A.M. (1980). Conduction-band structure of a ferromagnetic semiconductor. Phys. Rev. B.

[48] Lascaray J.P., Deruelle M.C.D., Coquillat D. (1987). Magnetization and magnetoreflectivity measurements in Zn_1−*x*_Mn_*x*_Te with 0.25 ≤ *x* ≤ 0.71. Phys. Rev. B.

[49] Lascaray J.P., Coquillat D., Deportes J., Bhattacharjee A.K. (1988). Zeeman splitting of exciton and magnetization in Cd_1−*x*_Mn_*x*_Te: Anomalous behavior at high *x*. Phys. Rev. B.

[50] Takahashi M., Kubo K. (2005). Limitation of mean field approximation for carrier states and ferromagnetism in diluted magnetic semiconductors. J. Phys. Soc. Jpn..

[51] Chattopadhyay A., Das Sarma S., Millis A.J. (2001). Transition temperature of ferromagnetic semiconductors: A dynamical mean field study. Phys. Rev. Lett..

[52] Yagi M., Kayanuma Y. (2002). Theory for carrier-induced ferromagnetism in diluted magnetic semiconductors. J. Phys. Soc. Jpn..

[53] Calderón M.J., Gomez-Santos G., Brey L. (2002). Impurity-semiconductor band hybridization effects on the critical temperature of diluted magnetic semiconductors. Phys. Rev. B.

[54] Zener C. (1951). Interaction between the *d*-shells in the transition metals. II. Ferromagnetic compounds of manganese with perovskite structure. Phys. Rev..

[55] Linnarsson M., Janzen E., Monemar B., Kleverman M., Thilderkvist A. (1997). Electronic structure of the GaAs:MnGa center. Phys. Rev. B.

[56] Iye Y., Oiwa A., Endo A., Katsumoto S., Matsukura F., Shen A., Ohno H., Munekata H. (1999). Metal-insulator transition and magnetotransport in III-V compound diluted magnetic semiconductors. Mater. Sci. Eng. B.

[57] Ohldag H., Solinus V., Hillebrecht F.U., Goedkoop J.B., Finazzi M., Matsukura F., Ohno H. (2000). Magnetic moment of Mn in the ferromagnetic semiconductor (Ga_0.98_Mn_0.02_)As. Appl. Phys. Lett..

[58] Okabayashi J., Kimura A., Mizokawa T., Fujimori A., Hayashi T., Tanaka M. (1999). Mn 3*d* partial density of states in Ga_1−*x*_Mn_*x*_As studied by resonant photoemission spectroscopy. Phys. Rev. B.

[59] Okabayashi J., Kimura A., Rader O., Mizokawa T., Fujimori A., Hayashi T., Tanaka M. (2001). Angle-resolved photoemission study of Ga_1−*x*_Mn_*x*_As. Phys. Rev. B.

[60] Okabayashi J., Kimura A., Rader O., Mizokawa T., Fujimori A., Hayashi T., Tanaka M. (1998). Core-level photoemission study of Ga_1−*x*_Mn_*x*_As. Phys. Rev. B.

[61] Ishiwata Y., Watanabe M., Eguchi R., Takeuchi T., Harada Y., Chainani A., Shin S., Hayashi T., Hashimoto Y., Katsumoto S., Iye Y. (2002). Manganese concentration and low-temperature annealing dependence of Ga_1−*x*_Mn_*x*_As by x-ray absorption spectroscopy. Phys. Rev. B.

[62] Park J.H., Kwon S.K., Min B.I. (2001). Electronic structures of III-V based ferromagnetic semiconductors: half-metallic phase. Physica B.

[63] Shirai M., Ogawa T., Kitagawa I., Suzuki N. (1998). Band structures of zinc-blende-type MnAs and (MnAs)_1_(GaAs)_1_ superlattice. J. Magn. Magn. Mater..

[64] Szczytko J., Mac W., Twardowski A., Matsukura F., Ohno H. (1999). Antiferromagnetic *p*-*d* exchange in ferromagnetic Ga_1−*x*_Mn_*x*_As epilayers. Phys. Rev. B.

[65] Ando K., Hayashi T., Tanaka M., Twardowski A. (1998). Magneto-optic effect of the ferromagnetic diluted magnetic semiconductor Ga_1−*x*_Mn_*x*_As. J. Appl. Phys..

[66] Okabayashi J., Mizokawa T., Sarma D.D., Fujimori A., Slupinski T., Oiwa A., Munekata H. (2002). Electronic structure of In_1−*x*_Mn_*x*_As studied by photoemission spectroscopy: Comparison with Ga_1−*x*_Mn_*x*_As. Phys. Rev. B.

[67] Hayashi T., Hashimoto Y., Katsumoto S., Iye Y. (2001). Effect of low-temperature annealing on transport and magnetism of diluted magnetic semiconductor (Ga,Mn)As. Appl. Phys. Lett..

[68] Yu K.M., Walukiewicz W., Wojtowicz T., Kuryliszyn I., Liu X., Sasaki Y., Furdyna J.K. (2002). Effect of the location of Mn sites in ferromagnetic Ga_1−*x*_Mn_*x*_As on its Curie temperature. Phys. Rev. B.

[69] Korzhavyi P.A., Abrikosov I.A., Smirnova E.A., Bergqvist L., Mohn P., Mathieu R., Svedlindh P., Sadowski J., Isaev E.I., Vekilov Yu.Kh., Eriksson O. (2002). Defect-induced magnetic structure in (Ga_1−*x*_Mn_*x*_)As. Phys. Rev. Lett..

[70] Timm C. (2003). Disorder effects in diluted magnetic semiconductors. J. Phys-Condens. Matter.

[71] Okabayashi J., Kimura A., Rader O., Mizokawa T., Fujimori A., Hayashi T., Tanaka M. (2001). Electronic structure of GaMnAs studied by angle-resolved photoemission spectroscopy. Physica E.

[72] Oiwa A., Katsumoto S., Endo A., Hirakawa M., Iye M., Ohno H., Matsukura F., Shen A., Sugawara Y. (1997). Nonmetal-metal-nonmetal transition and large negative magnetoresistance in (Ga,Mn)As/GaAs. Solid State Commun..

[73] Oiwa A., Katsumoto S., Endo A., Hirakawa M., Iye M., Matsukura F., Shen A., Sugawara Y., Ohno H. (1998). Low-temperature conduction and giant negative magnetoresistance in III-V-based diluted magnetic semiconductor. Physica B.

[74] Matsukura F., Ohno H., Shen A., Sugawara Y. (1998). Transport properties and origin of ferromagnetism in (Ga,Mn)As. Phys. Rev. B.

[75] Hirakawa K., Katsumoto S., Hayashi T., Hashimoto Y., Iye Y. (2002). Double-exchange-like interaction in Ga_1−*x*_Mn_*x*_As investigated by infrared absorption spectroscopy. Phys. Rev. B.

[76] Katsumoto S., Hayashi T., Hashimoto Y., Iye Y., Ishiwata Y., Watanabe M., Eguchi R., Takeuchi T., Harada Y., Shin S., Hirakawa K. (2001). Magnetism and metal-insulator transition in III-V based diluted magnetic semiconductors. Mater. Sci. Eng..

[77] Akai H. (1998). Ferromagnetism and its stability in the diluted magnetic semiconductor (In, Mn)As. Phys. Rev. Lett..

[78] Szczytko J., Twardowski A., Świa̧tek K., Palczewska M., Tanaka M., Hayashi T., Ando K. (1999). Mn impurity in Ga_1−*x*_Mn_*x*_As epilayers. Phys. Rev. B.

[79] Szczytko J., Mac M., Stachow A., Twardowski A., Becla P., Tworzydlo J. (1996). The *s*,*p*-*d* exchange interaction in GaAs heavily doped with Mn. Solid State Commun..

[80] Kagami K., Takahashi M., Yasuda C., Kubo K. (2006). Theory of diluted magnetic semiconductors: A minimum model. Sci. Technol. Adv. Mater..

[81] Nazir S., Ikram N., Tanveer M., Shaukat A., Saeed Y., Reshak A.H. (2009). Spin-polarized structural, electronic, and magnetic properties of diluted magnetic semiconductors Cd_1−*x*_Mn_*x*_S and Cd_1−*x*_Mn_*x*_Se in zinc blende phase. J. Phys. Chem. A.

[82] Sato K., Katayama-Yoshida H. (2002). First-principles materials design for semiconductor spintronics. Semicond. Sci. Technol..

[83] Sakai O., Suzuki S., Nishizawa K. (2001). Study on the magnetic and transport properties of low density carrier ferromagnetic semiconductors. J. Phys. Soc. Jpn..

[84] Ohe J., Tomoda Y., Bulut N., Arita R., Nakamura K., Maekawa S. (2009). Combined approach of density function theory and quantum Monte Carlo method to electron correlation in diluted magnetic semiconductors. J. Phys. Soc. Jpn..

[85] Takahashi M., Mitsui K., Umehara M. (1993). Conduction-electron states in ferromagnetic semiconductors above the Curie temperature. Phys. Rev. B.

[86] Takahashi M., Mitsui K., Umehara M. (1995). Multiple-scattering approach to the *s*-*f* model in ferromagnetic semiconductors above the Curie temperature. Phys. Rev. B.

[87] Takahashi M., Kasuya T. (1983). A theory for a self-trapped molecular magnetic polaron in ferromagnetic semiconductor. J. Phys. Soc. Jpn..

[88] Takahashi M., Kasuya T. (1983). Effect of electron-phonon interaction in the magnetic polaron. J. Phys. Soc. Jpn..

[89] Edwards D.M., Green A.C.M., Kubo K. (1999). Electronic structure and resistivity of the double exchange model. J. Phys-Condens. Matter.

[90] Matsunaka D., Kasai H., Dino W.A., Nakanishi H. (2004). Dynamical cluster approximation in disordered systems with magnetic impurities. J. Phys. Soc. Jpn..

